# Profiling trial burden and patients’ attitudes to improve clinical research in epidermolysis bullosa

**DOI:** 10.1186/s13023-020-01443-3

**Published:** 2020-07-10

**Authors:** Christine Prodinger, Anja Diem, Katherina Ude-Schoder, Josefina Piñón-Hofbauer, Sophie Kitzmueller, Johann W. Bauer, Martin Laimer

**Affiliations:** 1 Department of Dermatology and Allergology, University Hospital of the Paracelsus Medical University Salzburg, Muellner Hauptstrasse 48, 5020 Salzburg, Austria; 2 EB House Austria, Department of Dermatology and Allergology, University Hospital of the Paracelsus Medical University Salzburg, 5020 Salzburg, Austria; 3 EB House Austria, Research Program for Molecular Therapy of Genodermatoses, Department of Dermatology and Allergology, University Hospital of the Paracelsus Medical University Salzburg, 5020 Salzburg, Austria

**Keywords:** Epidermolysis bullosa, Clinical trial, Rare disease, Recruitment failures, Challenges for trial design

## Abstract

**Background:**

Epidermolysis bullosa (EB) comprises inherited mechanobullous dermatoses with considerable morbidity and mortality. While current treatments are symptomatic, a growing number of innovative therapeutic compounds are evaluated in clinical trials. Clinical research in rare diseases like EB, however, faces many challenges, including sample size requirements and recruitment failures. The objective of this study was to determine attitudes of EB patients towards clinical research and trial participation as well as the assessment of contextual motivating and discouraging factors in an effort to support patient-centered RD trial designing.

**Methods:**

A 53-items questionnaire was handed over to EB patients (of all types and ages) in contact with the EB House Austria, a designated national center of expertise for EB care. Main categories included level of interest in and personal knowledge about clinical studies, pros/cons for participation and extent of individual expenses considered acceptable for participation in a clinical study. Descriptive subgroup analysis was calculated with SPSS 20.0 and Microsoft Excel.

**Results:**

Thirty-six individuals (mean age 25.7 years), diagnosed for recessive dystrophic EB (36.1%), EB simplex (33.4%), junctional EB (8.3%), dominant dystrophic EB (2.8%) and acral peeling syndrome (2.8%) participated. Motivation for participation in and the desire to increase personal knowledge about clinical trials were (outmost) high in 57.2 and 66.7%, respectively. Altruism was the major motivating factor, followed by hope that alleviation of the own symptoms can be achieved. The greatest hurdle was travel distance, followed by concerns about possible adverse reactions. Patients diagnosed for severe subgroups (RDEB, JEB) were more impaired by the extent of scheduled invasive investigations and possible adverse reactions of the study medication. Patients with generally milder EB forms and older patients were accepting more frequent outpatient study visits, blood takes, skin biopsies and inpatient admissions in association with trial participation.

**Conclusions:**

This study provides additional indications to better determine and address attitudes towards clinical research among EB patients as well as guidance to improve clinical trial protocols for patient centricity.

## Background

Epidermolysis bullosa (EB) comprises a rare heterogeneous group of genodermatoses characterized by hyperfragility of epithelialized tissues to mechanical forces. Clinical hallmarks include blisters, erosions, atrophy and scarring of skin and mucosal membranes. EB is caused by mutations in several genes involved in the maintenance of intraepidermal and dermoepidermal structural as well as functional integrity [1]. Epigenetic, biochemical and environmental factors additionally modulate the considerably broad phenotypic spectrum of EB, e.g. by trauma-induced activation and chronification of inflammatory cascades leading to tissue remodeling. Especially in the severe subtypes of junctional and recessive dystrophic EB, morbidity and mortality are high due to generalized skin and mucosal involvement as well as primary and secondary extracutaneous manifestations, making EB a systemic disease of high burden [1–3].

Current treatment strategies are primarily symptom-orientated and supportive, thereby defining a high unmet medical need for a critical portion of EB patients. Progress in molecular research has enlightened our knowledge about pathogenic traits in EB and provides targets of translational therapeutic potential. The number of innovative local or systemic treatment modalities is constantly growing, including approaches of protein, cell and gene therapy as well as symptom-relieving therapies targeting key mediators of aberrant molecular pathways [4]. In addition there is a steady increase in the number of investigational products, which are currently being tested in clinical trials [5–7].

Clinical research investigations are an indispensable precondition for proving the efficacy, safety and benefit-to-risk ratio of new treatments. However, trials for rare diseases (RD) like EB pose several challenges (Table 1) [28, 29]. Recruitment of the right patients in adequate numbers in a reasonable time-frame has been recognized as one of the biggest challenges, reflecting an intrinsically small number of candidates accessible within a feasible catchment area that are both, inclined as well as eligible based on their disease profiles and health status [30, 31]. On the other hand, patient-centric trial design with clinically meaningful endpoints and valid outcome measures is supposed to be a key measure to optimize trail recruiting and adherence. Faster enrollment and fewer drop outs also help to reduce expenses in inherently cost-sensitive RD research.

**Table 1:** Example of challenges and solution approaches for RD trials [8–27]

**Main challenges to RD research**	
**Disease characteristics**	
**Target population**	• Small number of patients • Eligible patients often geographically dispersed
**Heterogeneity** of disease and diseased study cohort	• Many genotypes and phenotypes; inconsistent genotype-phenotype correlations; improper diagnostics • Soft inclusion criteria to foster recruitment • Enhanced degree of random imbalance in covariates in small study samples ➔ limited generalizability and applicability of RD clinical trial results for real life
**Ethical issues**	• Concerns to conduct research in children who, however, are predominantly affected and at risk to develop early, potentially irreversible complications, thus benefit most from preventive therapeutics
**Patient perspectives**	
**Travel burden**	• Limited number of trial sites, complex medical problems, disease burden and health condition affecting transportability
**Financial burden for patients**	• Travel, accommodation, dependent care, off-work time, family/caregivers‘commitments
**Time consuming**	• Daily routine for (additional) dressing changes, patient diaries, photo documentation • Study visits at short intervals in addition to standard/routine clinical appointments
**Additional clinical tests**	• Invasive interventions on vulnerable skin e.g. blood tests, biopsies, additional dressing changes
**Higher risks**	• Generated evidence on safety and/or efficacy from clinical trials in small (adult) populations limited; • Attempts to reduce risks may increase complexity of clinical trials with coincident declining numbers of eligible and recruited patients per site
**Trial design/planning, study protocol**	
**Limited data**	• Limited knowledge on pathogenic disease traits, potential therapeutic targets and natural course; lack of knowledge on types and timing of outcomes; little background research to support clinical trial planning ➔ • Difficulties to identify key milestones; estimate expected effect size; calculate number of probands; define appropriate study length, clinical rating scales and suitable clinical trial endpoints
**Small sample size**	• Restricted replication and limited statistical power; limited acceptable evidence of efficacy; especially slight or moderate changes hardly reach statistical significance
**Outcome measures, endpoints**	• Determination of feasible, appropriate, well-defined, reliably measurable parameters that are relevant to patient, observable within a reasonable timeframe, sensitive to intervention • Complex endpoints reduce number of centers able to participate in trial
**Restrictive inclusion/exclusion criteria**	• Stringency usually enables a more uniform group of participants which is especially relevant in highly heterogeneous diseases/disease populations like in EB • Account for reduced variability and increased validity/statistical power/significance in trials with a small number of participants • Stringency may create (younger and healthier) trial population that is not representative of the population with the given disease (real life data)
**Complex safety testing**	• Required for cellular and molecular therapies • Needed to be tailored to particular types of individual patients
**Longer study periods**	• Slower enrollment due to fewer patients; more time necessary to capture meaningful data; lack of precedent, often “first in class” drugs; increased development costs, not expected to make huge revenues once drugs come to market due to small consumer base
**Concurrently recruiting trials**	• (Internal) competition for a small number of eligible patients
**Administrative burden / costs**	
**Administrative and logistical efforts**	• Small number of geographically dispersed patients and specialist centres • Multinational trials logistically difficult to conduct and costly (differences in regulatory and ethical requirements; hurdles of international contracting, insurance and liability laws; additional means of communication and translation; national cost variation; language and cultural barriers, inherent differences in healthcare systems, different standards for diagnostics and of care; variable availability of treatment options, funding and research culture; risk of increased heterogeneity of patient population due to genetic (subsets) or environmental factors
**Approaches to overcome obstacles in RD research**	
**Addressing disease characteristics**	
**Disease registries**	• Encourage and facilitate clinical research (correlation of complex genotype/phenotype relationships; determination of epidemiological and prognostic markers to identify and comprehensively characterize disease traits; enable accurate prenatal/preimplantation/predictive diagnosis, prognostication and determination of recurrence risks)
**Natural history / observational studies**	• Increase knowledge about pathogenic disease traits and natural course
**Addressing trial design/planning, study protocol**	
**Statistical analysis plans**	• Rigorous sample size planning and statistical analysis to precisely define probabilities of a false positive and false negative error in conclusions
**Multi-centre trials**	• Increase sample size through (international) recruiting, collaboration and networking • For lower costs and tighter timelines, prevalence of an illness should determine where a site is activated
**Research networks**	• Identification and cross-linking of specialized centers and disease specific registries • Data/knowledge/expertise sharing, dissemination of information among experts (standardized registries with international interoperability, inventories, partnership with patient organizations) to boost recruitment, trial feasibility and international research collaboration
**Protocol discussion**	• Assembly of a study review panel comprising patients, EB physicians, nurses, researchers, statisticians with assessment of appropriate/feasible rationale, methodology, endpoints/outcome measures, inclusion/exclusion criteria
**Patient centricity**	• Patients to co-decide on clinically meaningful endpoints, patient-relevant outcome measures, surmountable trial burden, study portfolio and amendments to meet patients’ demands and priorities, thereby fostering faster recruiting/enrollment, reduced complexity and drop out rates, faster drugs marketing • Costs of gathering such patient input on protocol design are additionally reported to be relatively low compared to the potential benefits
**Ethical principles**	• Distinct consideration of disease severity and adequacy of alternative treatments especially in paediatric population
**Pharmacovigilance regulations**	• Evaluation and discussion of acceptable trial burden for patients with authorities and sponsors
**Alternative clinical trial designs**	• May decrease necessary sample size; increase information obtained from each enrolled subject, trial acceptability and enhance patient enrollment
**Regulatory and legal issues**	• Global regulatory strategy and global operational execution; harmonization of regulatory and funder requirements and institutional policies to reduce complexity
**Addressing patient perspectives / recruitment**	
**Electronic patient recruitment**	• Exploit impact of social media; patient communities homepage; messaging or telephone reminders to increase awareness • Access to registry data and referral networks
**Placebo control**	• Allowing standard of care treatment instead of placebo control; alternative clinical trial designs (e.g. cross-over); minimize the use of placebo (e.g. allocation ratio)
**Site support**	• Concierge service; minimal waiting time; all assessments within local facility; comfortable environment
**Transparency**	• Transparent practices: availability and communication of clinical trial results for/to patients
**Patient education**	• Comprehensible, age-adapted patient education and information material, clear consent forms • Use of various media formats to provide key messages and outreach materials: videos, workshops/webinars, websites, newsletters, paper-handouts • Education on reliable sources that demonstrate a close collaboration between medical experts, sponsors, academia, regulatory agencies, patient groups • Layperson’s summaries on ongoing and scheduled trials via homepage, emails and press releases • Explaining thoroughly and objectively informed consent procedures; giving realistic expectations on the basis of preclinical safety and toxicity testing to address therapeutic misconceptions (“new is not always better”; misconstruction of research as therapy); clarification about the study purpose including production of generalizable knowledge with potentially no direct benefit • Involvement of trial experienced patients serving as authentic promoters
**Travel burden**	
**Facilitated travelling**	• Comfortable lodgings and logistical support (concierge-level service for transportation and booking)
**Home healthcare services**	• Home-based support and delivery of study medication carried out by homecare health practitioners, if applicable (e.g. for drug infusions, blood draws, minimally-invasive tests including pharmacokinetic sampling)
**Flexible study design**	• Critical review of study protocols for feasible frequency of on-site visits; flexible slots for on-site visits (including assessment schedules with early, late or weekend appointments); alternate assignment of participants to data collection time points to reduce sample collection burden
**Alternative clinical trial designs**	• e.g. shared care sites, “hub and spoke” trial design (major procedures performed at the main study site; minor procedures happen on local sites); cross-over design, series of n-of-trials; response-adaptive study design; factorial designs, etc.
**Cost reduction**	• Upfront payments or reimbursement from study account of trial-related added expenses, especially travel costs and accommodation for patients and caregivers
**Mobile and web-based technology**	• For data collection

Against this background, we conducted a survey among patients and caregivers in contact with the EB House Austria, a designated national center of expertise for EB care, with the aim to determine attitudes towards clinical research and trial participation, to assess motivating and discouraging factors in the context of disease burden, age and personal clinical research experience as well as to provide additional indications to improve patient-centricity of trial designs in EB.

## Methods

This survey was conducted among patients of the EB House Austria using an anonymous, self-created and not validated questionnaire. The study was approved by an institutional review board of the patient advocacy group DEBRA Austria. Participants were recruited irrespectively of subtype and age during an 8-month enrollment period (12/2018 to 07/2019). To raise participation, the questionnaire was introduced at patients’ visits in the EB House, during the annual meetings of DEBRA Austria and DEBRA Italy, and was sent to subscribed DEBRA-members with anonymous return envelopes. Caregivers were asked to complete the questionnaire on behalf of affected minors/underage children unable to respond adequately.

The questionnaire was designed using layman’s language (either in German or in Italian) and specified response options along a 5-point Likert scale, graded from 1 (not at all present/not at all important) to 5 (very high/outmost important). In addition, three open-ended questions, one multiple answer and 8 text entry questions were inquired. Options to include additional comments were provided throughout the questionnaire.

In total 53 questions were designed based on the experience of the EB House study team and a review of literature on trial burden [32–35]. They were divided into six categories: demographic data (4 questions), general health and quality of life (4 questions), level of self-reported interest to participate in and personal knowledge about clinical studies (8 questions), pros (16 questions) and cons (14 questions) for participation in a clinical trial, and extent of individual expenses considered to be acceptable for participation in a clinical study (7 questions).

Descriptive statistics, including percentages of total responses and sub-group analyses to identify potential differences, were calculated using SPSS 20.0 and Microsoft Excel. The Likert scala points 1 and 2 as well as 4 and 5 were combined for analysis. For reliability analysis, Cronbach’s alpha was calculated to assess the internal consistency using eight questions defining a positive attitude towards clinical studies as a subscale as well as inter-item correlation. Subgroups of generally milder (EBS, APS,^1^ DDEB) against commonly more severe EB types (JEB, RDEB), younger (< 18 years) against older (≥18 years) patients, participants with against those without trial experience, responders with positive against those with negative attitudes towards participation in a clinical trial as well as read-outs of self- versus parent proxy-reports were defined for discriminant analyses. Single items with missing data entries were censored from analysis. A chi-square test of independence or, in case of a 2 × 2 contingency table a Fisher’s exact test, were performed to examine the relation between subgroups and responses to the items and to compute exact *p*-values for each cell in a contingency table. In addition, Spearman’s correlation coefficient test was used. The statistical significance level was set at *p* < 0.05 (two-tailed for chi square, one tailed for Fisher exact test) for all analyses. Mean values were calculated from arguments pro and against study participation in order to find out the rank of importance.

## Results

### Patient characteristics

A total of *n* = 36 questionnaires were eligible for analysis. Corresponding patients’ characteristics are shown in Table 2. 38.9% (14/36) of participants had been diagnosed for a milder EB type and 44.4% (16/36) for a severe EB type. Notably, this categorization was based on a formal classification according to the EB subtype without individual clinical scoring [1]. Among the subcohort aged younger than 18 years (33.3% [*n* = 12/36]), data acquisition was based on parent proxy-reports in 50% (6/12).

**Table 2:** Demographic data of the study cohort (*n* = 36)

Category	Patients (n)	Percentage (%)
**Sex**		
Female	19	52.8
Male	15	41.7
n/a	2	5.5
**Age**		
Mean age	25.7 years (range 5–80)	
< 18a	12	33.3
≥ 18a	19	52.8
Not specified	5	13.9
**Country of origin**		
Austria	17	47.2
Germany	10	27.8
Italy	5	13.9
n/a	4	11.1
**EB Subtypes**		
Mild		
EBS	12	33.3
Acral peeling syndrome	1	2.8
DDEB	1	2.8
Severe		
JEB	3	8.3
RDEB	13	36.1
n/a	6	16.7
**Participation in previous clinical trials**^**a**^		
Yes^**a**^	27^**a**^	75.0
No	9	25.0

*n/a* not available

^a^Participation in previous clinical trials was equated with the number of participants answering the question: “*For participants in previous / current studies: My willingness to persuade others (friends, family, patients) to participate in a clinical study is ..”*

### Quality of life and health condition

**Fig. 1:**
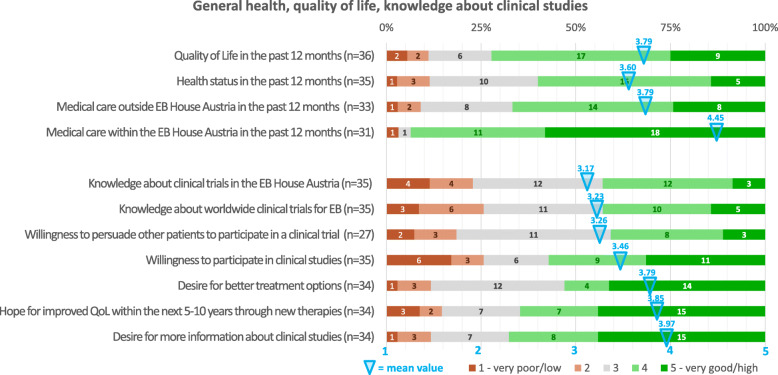
General health, quality of life, knowledge about clinical studies. Graphical presentation of patients’ answers (in percentage) to part one of the survey which includes questions addressing their general health, quality of life and knowledge about clinical studies. Mean values (Likert scale graded from 1 to 5) are crayoned. The numbers in the columns represent respondents for each option

A self-rated “excellent” or “good” quality of life and health condition in the last 12 months was stated by 72.2% (26/36) and 60.0% (21/35), respectively (mean 3.79 (SD 1.07) and 3.60 (SD 0.95) points) (Fig. 1). Upon stratification, the item “health condition” was rated (very) good in 84.6% (11/13) of patients in the milder group compared to 50.0% [8/16] in the severe group (*p* = 0.11) (Fig. S2).

### Motivation for trial participation

The motivation to participate in a clinical trial was (outmost) high in 57.1% (20/35) of all participants (mild types: 64.3% [9/14], severe types: 53.3% [8/15]) (Fig. 1, S2). 75% (9/12) of younger patients expressed to be (outmost) highly motivated to participate in a trial compared to 44.4% (8/18) of the older patients (*p* = 0.10) (Fig. S3). Likewise, the younger subgroup was significantly less averse to participate (8.3% [1/12] versus (vs) 44.4% [8/18], *p* = 0.040). Trial participation was further favored by 59.3% (16/27) of patients with previous study experience compared to 50% (4/8) of patients without (*p* = 0.473).

### Symptomatic relief defining study successfulness

Participants were asked to note down the percentage of symptomatic relief in the major individual complaint (in this cohort pruritus (36.4% [4/11]), blistering (27.3% [3/11]), pain (27.3% [3/11]) that, in the patients’ judgement, would suffice to consider a study successful. Four individuals (1 with mild EB type, 1 severe EB type, 2 without indicated subtype) replied a 50% reduction as sufficient and 2 patients (both severe EB type) 30%. The significance of these results is considerably limited due to the low response rates in these text-entry questions.

### Desire for knowledge and information

The desire to increase the personal knowledge about clinical studies in general as well as to receive more information on the locally available study portfolio was (outmost) high in 67.6% (23/34) of all participants (Fig. 1). This item significantly correlated with the expression of high hopes that new therapies will improve the personal quality of life (qol) within the next 5–10 year (64.7% [22/34]; *r* = 0.626; *p* < 0,001). 33.3% (4/12) of younger patients considered themselves to be less well informed, compared to 61.1% (11/19) of older patients (*p* = 0.132) (Fig. S3). Participants named EB newsletter (*https://www.debra-austria.org/newsletter*; 63.9% [23/36]), patient advocacy group (61.1% [22/36]), the EB House Austria (47.2% [17/36], internet (41.7% [15/36]) and other patients (33.3% [12/36] as major sources of knowledge about clinical trials (Fig. S5).

### Arguments for trial participation (Fig. 2)

Altruism was identified as the major driving force to personally participate in a clinical trial. For 87.5% (28/32) of our cohort, an (outmost) important reason to take part in a clinical trial was the hope for better treatments for other EB patients in future (mean 4.59, SD 0.79) and for 68.8% (22/32) that their participation contributes to an increase in knowledge about the disease (mean 3.95, SD 1.23). Alleviation of own symptoms was a key motif for 77.4% (24/31) of responders (mean 4.29, SD 1.07).

**Fig. 2:**
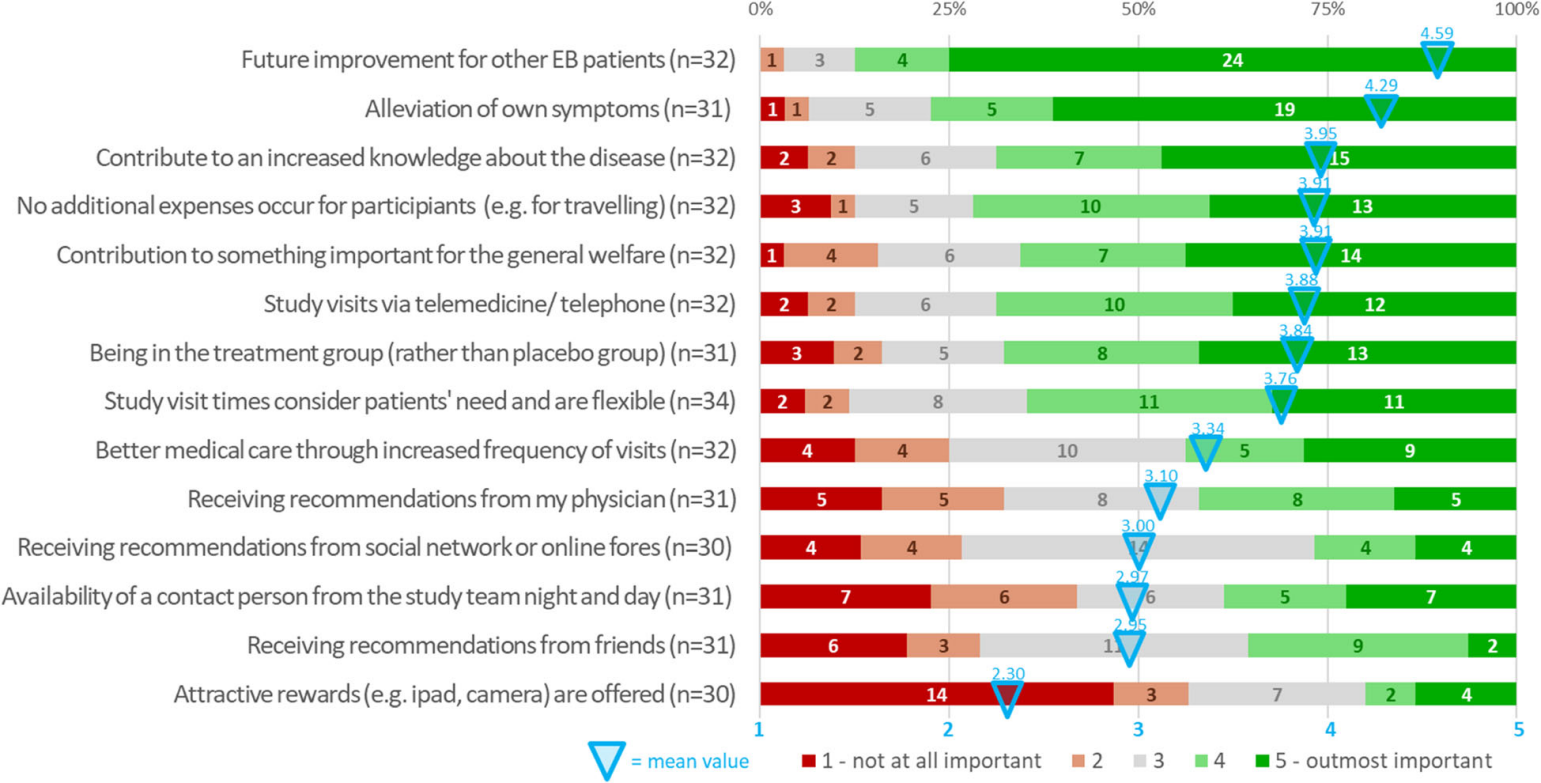
Arguments for participation in a clinical trial. Graphical representation of patients’ answers (in percentage) to part two of the survey that comprises questions addressing the main arguments for participation in a clinical trial. The list is sorted by mean values (crayoned in blue; according to a Likert scale graded from 1 to 5) in descending order. The numbers in the columns represent respondents for each option

### Arguments against trial participation (Fig. 3)

Travel distance to reach the study center turned out to be the most prominent hurdle (mean 3.65, SD 1.65) that was (outmost) relevant to 67.7% (23/34) of patients. The second most important reason against participating were concerns about the scope of possible adverse reactions or unknown risks of the study medication (45.5% (15/33) mean 3.36; SD 1.41).

**Fig. 3:**
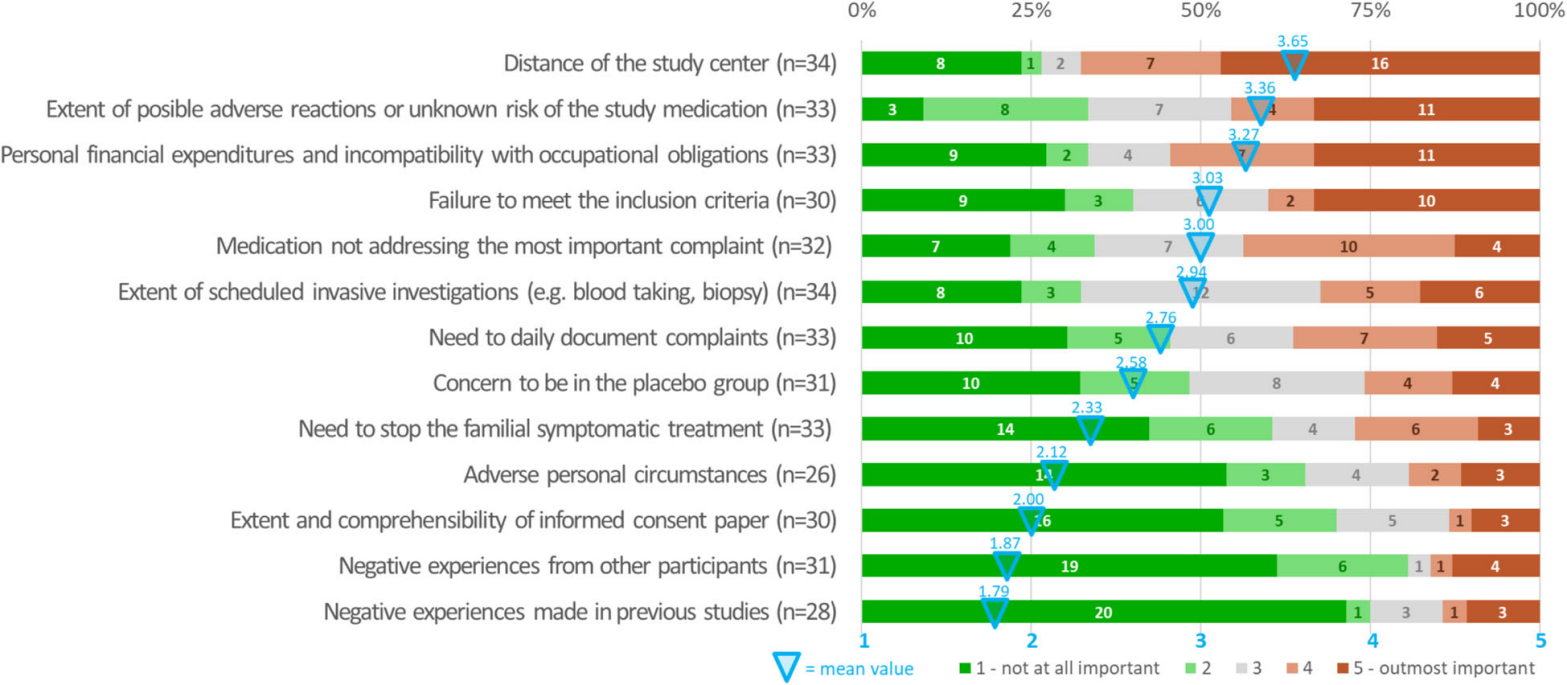
Arguments against participation in a clinical trial. Graphical representation of patients’ answers (in percentage) to part three of the survey that comprises questions addressing the main obstacles for participation in a clinical trial. The list is sorted by mean values (crayoned in blue; according to a Likert scale graded from 1 to 5) in descending order. The numbers in the columns represent respondents for each option

### Additional subgroup stratifications

Subgroup analyses further revealed that, compared to milder EB phenotypes, *severely affected patients* had a significantly higher “desire for better treatment options” ((outmost) high in 73.3% [11/15] compared to 30.8% [4/13], *p* = 0.030) (Fig. S2). In addition, they worried more about the “extent of scheduled invasive investigations” (46.7% [7/15] vs 7.7% [1/13], *p* = 0.029) as well as “extent of possible adverse reactions or unknown risk of the study medication” (53.3% [8/15] vs 23.1% [3/13] *p* = 0.106). Furthermore, “personal financial expenditures and incompatibility with occupational obligations” were considered in 73.3% (11/15) an important argument against participation in the severe subgroup (vs 41.7% [5/12] in the mild group, *p* = 0.102). (Fig. S2).

Patients with *milder EB types* reported a high desire for flexible study visit schedules ((outmost) important for 83.3% [10/12] vs 53.3% [8/15] in the severe group, *p* = 0.108) as well as telemedicine offers ((outmost) important for 90.9% [10/11] vs 46.7% [7/15] (*p* = 0.024)) (Fig. S2). High rated reasons for participation in this subgroup additionally were the contribution “to something important for the general welfare” (81.8% [9/11] vs 53.3% [8/15], *p* = 0.138) and “to increase knowledge about the disease” (81.8% [9/11] vs 60.0% [9/15], *p* = 0.226).

Compared to participants aged ≥18 years, the *younger subgroup* showed a significantly higher desire for better treatment options (in 75.0% [9/12] (outmost) high vs 35.3% [6/17], *p* = 0.041). They rated their health status excellent/very good in 83.3% [10/12] (vs 55.6% [10/18] in the older group, *p* = 0.117). Younger participants additionally reported the argument to “contribute to something important for the general welfare” to be significantly more important (*p* = 0.042), while “no additional expenses to occur alongside participation” were significantly more relevant for older patients (*p* = 0.010). The latter also considered adverse personal circumstances to be a significantly higher barrier for participation (*p* = 0.027) and were significantly more hampered by the “failure to meet the inclusion criteria” (62.5% [10/16] vs 10.0% [1/10], *p* = 0.011).

Patients expressing a *high or outmost high motivation to participate* in a clinical trial valued the following pro arguments higher, compared to patients with loath attitudes (low or no motivation): to “contribute to an increased knowledge about the disease” (89.5% [17/19] vs 28.6% [2/7], *p* = 0.006); “being in the treatment group (rather than placebo group)” (77.8% [14/18] vs 28.6% [2/7], *p* = 0.34); “study visit times consider patients’ need and are flexible” (85.0% [17/20] vs 28.6% [2/7], *p* = 0.011).

Otherwise, subgroup analyses revealed no significant results

### Extent of individual expenses

**Fig. 4:**
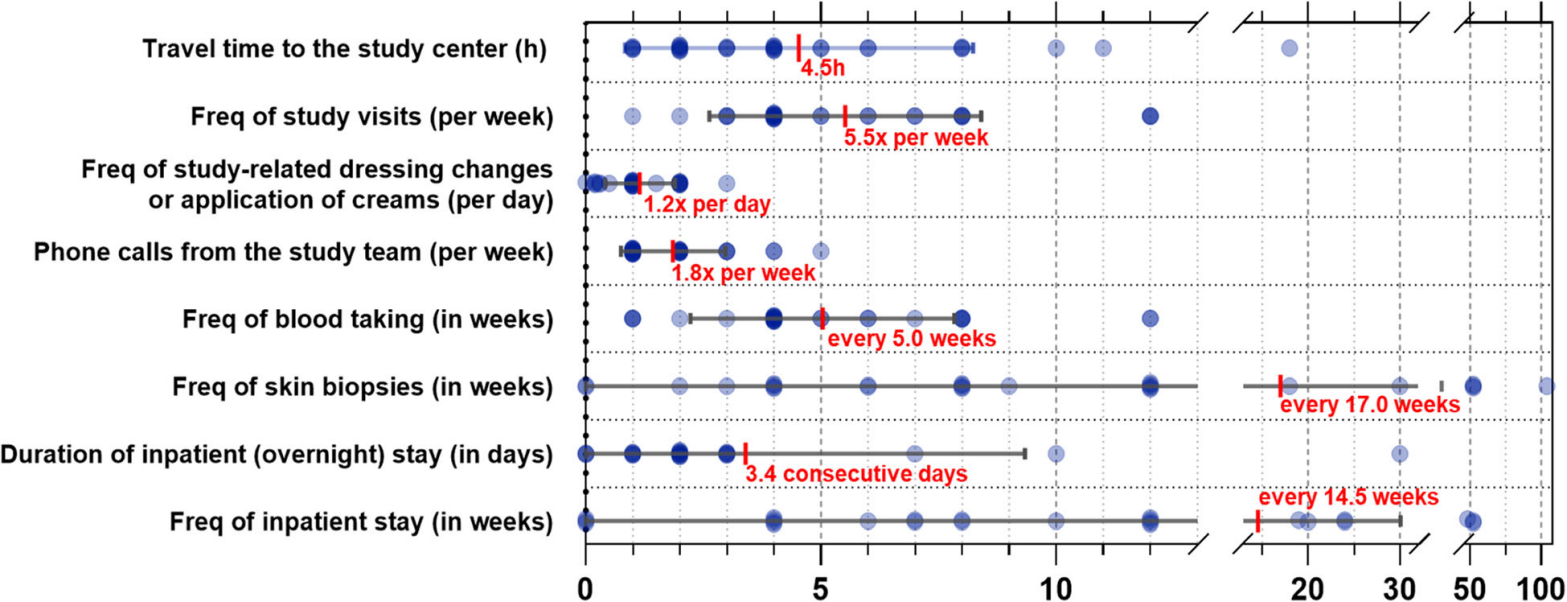
Extent of individual expenses considered acceptable for participation in a clinical study. The mean maximum travel time for regular outpatient or day-clinic visits at the study center (*n* = 30) was calculated to be 4.5 h (range 1-18 h, mean: mild 3.9 h, severe 5.71 h; younger 5.3 h, older 4.5 h). The maximally tolerated frequency of study visits (*n* = 29) was every 5.5 weeks (range 1–12 weeks) and of blood taking (*n* = 28) every 5.0 weeks (range every 1–12 weeks). Skin biopsies (*n* = 26) were considered to be taken not more often than every 17.0 weeks (range 4–104 weeks). Two patients (7.7%) stated to not allow this intervention at all (dots on the x-axis). Inpatient admission for 3.4 consecutive days (0–30 days; *n* = 25)) every 14.5 weeks (range 4–52; *n* = 24) was reported to be acceptable during the study period. According to this survey, a maximum of 1.2 dressing changes or applications of investigational topical treatments per day as well as 1.8 (range 1–5) study calls per week would be acceptable (*n* = 28)

Data on the extent of individual expenses considered acceptable for participation in a clinical study are illustrated in Fig. 4. Upon stratification, patients with generally milder EB forms as well as older patients were accepting more frequent outpatient study visits, blood takes, skin biopsies and inpatient admissions in comparison to individuals with more severe EB types and the younger subgroup. Responders with severe EB types and older participants would overall agree to stay longer at hospital. (Fig. S4).

#### Internal consistency

Cronbach’s alpha for internal consistency of questions addressing a positive attitude towards clinical studies reached an acceptable reliability of α = 0.78. Two questions addressing the same item (being in the treatment group versus concern to be in the placebo group) are positively correlated (r (34)=0.41, *p* = 0.029) and within the ideal range of inter-item correlation.

## Discussion

According to this survey, motivation for participation in and desire for knowledge and information about clinical trials is considerably high in our EB cohort. To exploit these opportunities for clinical research, patient information and education strategies are critical. Campaigning for potential participants has to accurately address individual expectations and attitudes (Table 1). For instance, parents of young children and adolescents generally not only have a higher interest in clinical trials but also higher expectations than older patients, who could have tempered their expectations, and may be looking for small improvements in symptoms [36].

Despite an obviously high level of self-reported motivation, recruitment failures, however, are common also at the EB House Austria. Even in general clinical research, about a third of phase 3 studies fail to meet recruitment targets and more than 50% of trials need to be extended to avoid being underpowered [37, 38]. Especially in RD research, profiling and addressing of patient-rated pros and cons for study participation are thus essential in an approach to counteract these difficulties. (Table 1).

In line with previous reports and other populations [32, 39], altruistic motifs were the most important reason for all EB patients of our cohort to participate in a clinical trial, followed by hope that alleviation of the own symptoms can be achieved (Fig. 2). Our data indicate that recommendations by physicians, social networks/online fora as well as incentives are less motivational. This somewhat contrasts to our finding that physicians and internet are among the important sources of information. (Fig. S5) It is also contrary to previous data identifying physicians’ recommendation to play a key role in patients’ beliefs about clinical trials and in their decision [34, 40–43]. Former studies likewise highlighted the relevance of social media. Close patient communities corresponding through these platforms are typical for rare diseases like EB. Remarkably, patients mentioned to discuss trial treatment in such fora, thereby potentially also hampering double blind standard of placebo controlled (‘breaking blinding’ or ‘unintentional unblinding’) [44]. Against this background, our results may reflect two separate dimensions: seeking and exploiting activities using various sources of information as well as individual decision-making based on the self-acquired information. They may, however, also be based on some reluctance of attending clinicians against clinical trials (with regard to, e.g. “allocation risk” to placebo; availability of similar, already marketed products; trial (protocol) burden; necessity to discontinue a well-accepted, somewhat successful and tolerated pre-treatment).

Travel distance to the study center was identified as the most important reason against trial participation in our population. In this context, physical impairments especially in patients with severe EB types may pose an insurmountable barrier in addition to increased time and financial investments. However, due to the rareness of the disease and geographic dispersion of potential subjects as well as a limited number of study centers with subsequently wide catchment areas, rather long travel routes to the study site are predetermined (in case of patients with regular contact to the EB House Austria up to 700 km) [32, 45]. Approaches to address travel burden and other motifs against trial participation are summarized in Table 1.

Subgroup analyses on motivators and demotivators showed that patients with severe EB types have a higher desire for better treatment options, which likely reflects a higher medical need. This cohort, which suffers from a generally higher disease burden and pronounced tissue hyperfragility, is also less amenable to accept invasive study investigations/interventions. A high trial burden likely impairs enrollment of severely affected individuals. Therefore, study plans should be evaluated to optimize protocols for recruitment, compliance and adherence (e.g. patient-centered study design permitting access to verum in a setting where valid efficient treatment is still beyond reach; frequency of on-site study visits; frequency and extent of invasive measures like biopsies and blood takes; appropriate flexibility in eligibility criteria) (Tab. 1). This, however, needs careful consideration and review of preclinical and available clinical data for justification and discussion with e.g. biostatisticians and regulatory authorities. In terms of patient-centered study endpoints, this survey suggests a symptomatic relief of not less than 30–50% in the participants’ major complaint would suffice to consider them a study targeting this symptom as successful. Although this impression is based on very limited data, trial designs (as well as patient education/information) may have to consider and address such remarkably high levels of claimed effectiveness with the intention to meet patients’ demands.

Responders suffering from severe EB variants also rated personal financial expenditures and incompatibility with occupational obligations to be cons of higher relevance that argue against trial enrollment. Again, daily life activities of this subgroup may be highly restricted (work, study or social commitments along with the large amount of time taken in daily routine for dressing changes and standard clinic appointments). In addition, caregivers are more occupied by home care. These conditions limit professional opportunities and financial standing due to low income and high expenditures for EB care. Additional transportation to the study site necessitating to take time off work as well as lodging cause additional costs hard to afford. (Table 1).

In contrast, the subgroup of patients with milder EB subtypes expressed a high desire for more flexible study visit schedules as well as telemedicine offers to facilitate trial participation. This may also reflect more occupational activity and thus obligations compared to severely affected participants.

In this context, it is noteworthy that our results on self-rated quality of life (qol) and health condition give the impression that the former is less dependent on disease severity, revealing an (excellent or) good qol irrespectively of EB subtype (that -if severe- typically show a chronic, progressive, debilitating and even life-threatening/−limiting course). This notion is consistent with previous studies in which patients with disabilities generally reported qol levels that are much higher than expected considering their objective condition [46–49]. These findings implicate a remarkable ability to adapt to discomfort and disease as well as the propensity to relate and compare personal well-being with other patients, but not healthy individuals. Notably, currently available, validated qol instruments may not accurately capture dimensions specific to EB [50–54]. Against this background, the item in this survey gives an impression about patients’ subjective wellbeing and satisfaction, although it does not adequately reflect the multidimensionality of measuring qol [55].

This study has significant limitations. As a single-center study and due to a confined number of participants, the significance and generalizability of the results are limited. A selection bias is related to the fact that all participants, including those contacted through the DEBRA Italy support group, volunteered. Thus, the study may have selected people with a more conscious commitment to deal with the disease, the EB House Austria and with clinical trials. In addition, patients who responded to this survey are most likely individuals who approve medical research and are interested in the pursuit of scientific knowledge. Thus, persons who do not enroll in clinical trials because they dislike or distrust the process (or purpose) of the clinical trials may be inadequately represented. The use of a hypothetical trial, though common in studies assessing the willingness to participate, may not elicit identical decision-making processes as would be found if patients were contemplating actual trial participation. Our results are additionally prone to reporting bias of participants, which have access to a highly developed health care system as well as to a designated center of expertise. Moreover, the questionnaire used is not validated and the population included in the study is heterogeneous as all EB types were eligible of which some subtypes are represented by only a single patient. A further limitation is that subgroup division into mild and severe disease was based solely on the formal diagnosis of the EB subtype but not on clinical scores (such as iscorEB [56], EBDASI [57] or BEBS score [58]), assessing the actual disease burden. Finally, in the subgroup of patients younger than 18 years, completion of the questionnaire may be significantly influenced by perspectives of caregivers.

## Conclusion

Despite significant limitations, this study provides additional indications to better determine and address attitudes towards clinical research among EB patients as well as guidance to optimize clinical trial protocols for patient centricity in EB as well as for other rare skin diseases.

## Supplementary information

**Additional file 1: Supplementary Fig. 1.** Patient questionnaire (translated into English, original version in German).

**Additional file 2: Supplementary Fig. 2a-c.** Subgroup results – mild versus severe. Graphical representation of subgroup responses referring to diseases severity (patients with mild EB versus patients with severe EB). The numbers in the columns represent respondents for each option. By combining Likert scala points 1 and 2 as well as 4 and 5 we identified significant relations between disease severity and responses to three questions (*): a) The “desire for better treatment options” was higher in the severe group (73.3% vs 30.8%, *p* = 0.030); b) “Study visits can be organized via telemedicine or telephone” is more important for the mild group (90.0% vs 46.7%, *p* = 0.024); c) The “extent of scheduled invasive investigations (e.g. blood taking, biopsy)” that is a more important argument against participation for the severe subgroup (46.7% vs 7.7%, 0 = 0.029). Arguments for and against participation in a study were sorted by the subgroup’s total mean values in descending order.

**Additional file 3: Supplementary Fig. 3a-c.** Subgroup results - young (0–17 years of age) versus old (≥18 years of age). Graphical representation of the responses of age-subgroups (patients 0–17 years and patients 18 years of age or older). The numbers in the columns represent respondents for each option. By combining Likert scala points 1 and 2 as well as 4 and 5, we found that younger patients had a significant higher desire for better treatment options (75.0% vs 35.3%, *p* = 0.041), were significantly less averse to participate (8.3% [1/12] vs 44.4% [8/18], *p* = 0.040) and rated “the failure to meet inclusion criteria” a significantly less important barrier (10.0% vs 62.5%, *p* = 0.011)(*).

**Additional file 4: Supplementary Fig. 4** Maximum extent of individual expenses considered acceptable for participation in a clinical trial. Graphical presentation of part four of the survey, asking for the extent of individual expenses considered acceptable for participation in a clinical study. Mean values of the four subgroups (mild (patients with mild EB type), severe (patients with severe EB type), young (< 18 years of age), old (18 years of age or older)) are indicated in different colors.

**Additional file 5: Supplementary Fig. 5.** Main sources of knowledge about clinical studies. The main sources of knowledge about clinical studies in this study cohort were the EB-newsletter (*https://www.debra-austria.org/newsletter*) 63.9%, *n* = 23), the patient groups DEBRA Austria and Italy (61.1%, *n* = 22), the EB House Salzburg (47.2%, *n* = 17), internet (41.7%, *n* = 15), and annual DEBRA Austria meetings (13.9%, *n* = 3). Three participants (8.3%) stated to have lacked any sources.

## Data Availability

The datasets generated during and/or analysed during the current study are not publicly available due to internal policies (limitations given by the hospital’s IT department) but are available from the corresponding author on reasonable request.

## Footnotes

1 The 2020 nomenclature and classification system of EB defines classical EB as the prototype of genetic disorders with skin fragility. Other disorders with skin fragility, including acral peeling syndrome, are reclassified as separate categories, i.e. so-called “EB-related disorders”. In these entities blisters are a minor part of the clinical presentation or are not seen owing to the very superficial skin cleavage [1].
