# Supplementary material for: Profiling trial burden and patients’ attitudes to improve clinical research in epidermolysis bullosa
Source: Orphanet J Rare Dis. 2020 Jul 10;15:182. doi: 10.1186/s13023-020-01443-3 (PMC7350741; doi:10.1186/s13023-020-01443-3)
Supplement: Supplementary file 1 — Additional file 1: Supplementary Fig. 1. Patient questionnaire (translated into English, original version in German). [file 13023_2020_1443_MOESM1_ESM.pdf]

UNIKLINIKUM SALZBURG
LANDESKRANKENHAUS

Landeskrankenhaus Salzburg
Universitätsklinik für Dermatologie der PMU
Vorstand: Univ.-Prof. Dr. Johann W. Bauer, MBA

eb Haus Austria. debra Hilfe für die Schmetterlingskinder.

EB-Haus Austria
Einheit für Klinische Forschung und EB Studienzentrum
Müllner Hauptstraße 48 |5020 Salzburg | Austria

QUESTIONNAIRE

CLINICAL STUDIES FOR EPIDERMOLYSIS BULLOSA

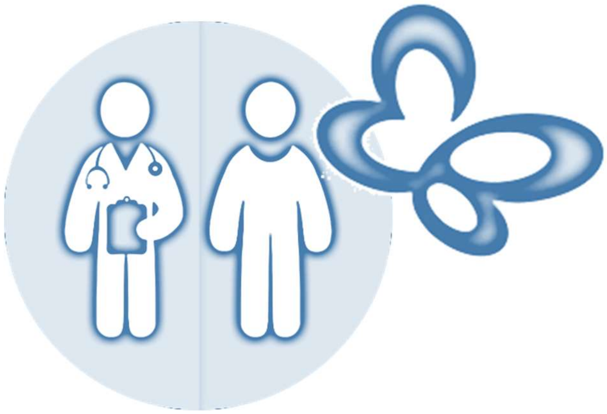

Home country ________________ Age ______ EB-subtype ______________ Gender _____________

GENERAL

How do you rate the following factors?

	Very poor 1	2	3	4	Excellent 5
My quality of life in the past 12 months was generally...	☐	☐	☐	☐	☐
My health status in the past 12 months was generally...	☐	☐	☐	☐	☐
My medical care outside the EB House Austria in the past 12 months was generally...	☐	☐	☐	☐	☐
My medical care within the EB House Austria in the past 12 months was generally...<br>Do you have any suggestions for improvement? ____	☐	☐	☐	☐	☐
My knowledge / information about current, worldwide clinical trials for new treatment options for EB is...	☐	☐	☐	☐	☐
My knowledge / information about current clinical studies in the EB House Austria is...	☐	☐	☐	☐	☐

From where do I retrieve information about (current) clinical EB studies?	☐ EB House Austria	☐ EB-newsletter	☐ Other patients	☐ Debra	☐ Internet	☐ Other: _____________

INFORMATION ABOUT CLINICAL STUDIES / RESEARCH

	Not at all present 1	2	3	4	Very high 5
My desire for more information about clinical studies in the EB Centre of Expertise is...	☐	☐	☐	☐	☐
My willingness to participate in clinical studies is...	☐	☐	☐	☐	☐
My desire for better treatment options is currently...	☐	☐	☐	☐	☐
My hope for improved quality of life within the next 5-10 years through new therapies is...	☐	☐	☐	☐	☐
For participants in previous / current studies: My willingness to persuade others (friends, family, patients) to participate in a clinical study study is ...	☐	☐	☐	☐	☐

ARGUMENTS FOR PARTICIPATING IN A CLINICAL TRIAL

How important are the following reasons for you to participate in a clinical study?

	Not important at all 1	2	3	4	Outmost important 5
I want to reach alleviation of my symptoms.	☐	☐	☐	☐	☐
I want to contribute to future improvements for other EB patients.	☐	☐	☐	☐	☐
I want to contribute to an increased knowledge about the disease.	☐	☐	☐	☐	☐
A participation in a study gives me more confidence since I receive better medical care due to increased frequency of study visits.	☐	☐	☐	☐	☐
I want to contribute to something important for the general welfare	☐	☐	☐	☐	☐
If I participate, I want to be sure that I receive treatment with active substance and that I am not in the placebo group (without active substance).	☐	☐	☐	☐	☐

Not important at all — Outmost important

	1	2	3	4	5
Study visits in the study center take into account my needs and are flexible, e.g. take place on weekends	☐	☐	☐	☐	☐
Study visits (except for start and end visits) are flexible and can be done via telephone and telemedicine (e.g. Skype) from at home	☐	☐	☐	☐	☐
Participation in the study does not cost me anything (i.e., replacement of travel and food costs).	☐	☐	☐	☐	☐
My physician recommends me a participation	☐	☐	☐	☐	☐
A participation in the study is recommended in the internet / social networks / EB fora	☐	☐	☐	☐	☐
The study is recommended by friends or other patients	☐	☐	☐	☐	☐
I receive attractive rewards for my participation (e.g. ipad / camera for televisits, that I can keep after the end of the study)	☐	☐	☐	☐	☐
A contact person from the study team is constantly available by phone (day and night).	☐	☐	☐	☐	☐

☐ I would rate a study as successful if it alleviates my most distressing complaint (____) by _______ %.

☐ Further important reasons for me to participate in a study are:

____

____

ARGUMENTS AGAINST PARTICIPATION IN A CLINICAL TRIAL

What are your main barriers for participating in a clinical trial?

No barrier at all — Outmost strong barrier

	1	2	3	4	5
The distance of the study center / travel expenses	☐	☐	☐	☐	☐
Personal financial expenditures or incompatibility with occupational / private obligations	☐	☐	☐	☐	☐
Extent of scheduled invasive investigations (e.g., blood taking, biopsy)	☐	☐	☐	☐	☐
Extent of possible adverse reactions or unknown risks of the study medication	☐	☐	☐	☐	☐
The expected effect of the study medication does not address the most important complaints of my disease	☐	☐	☐	☐	☐
Need to daily document the wound situation and complaints (e.g. study diary)	☐	☐	☐	☐	☐
I do not meet the inclusion criteria for the study (e.g. due to small wound area or too many comorbidities...)	☐	☐	☐	☐	☐
I'm worried about being in the placebo group (no active ingredient)	☐	☐	☐	☐	☐
I would have to stop my familial treatment (for example, ointments, bandages) during study participation.	☐	☐	☐	☐	☐
I have heard about negative experiences from other study participants.. (____which?)	☐	☐	☐	☐	☐
Extent and comprehensibility of the information materials (including informed consent paper) for a study.	☐	☐	☐	☐	☐
I have made negative experiences in previous studies: ____ (which?)	☐	☐	☐	☐	☐
Personal circumstances prevent me from participating ____ (which?)	☐	☐	☐	☐	☐

☐ Other important reasons for not participating in a study are:

____

____

MAXIMUM EXPENSES FOR A CLINICAL STUDY

Which of the following expenses in association with a study would I be willing to accept at the maximum, if the study medication could alleviate my main complaint (s)?

Travel time to the study center:	maximum ______ hours
Frequency of study visits:	maximum 1 study visit in _________ weeks
Frequency of blood taking:	maximum 1 blood take in ________ weeks
Frequency of skin biopsies:	maximum 1 skin biopsy in ________ weeks
Inpatient stay (with overnight stay)	maximum _______ days, every ________ weeks
Frequency of study-related dressing changes or creaming:	maximum __________ per day / per week *(bitte eine der beiden Möglichkeiten durchstreichen)*
Phone calls from the study team	maximum ___________ per week

Would you like to tell us something else about clinical trials?

____

____

____

____

____

____

____

____

____

____

____

____

____

Thank you for completing this questionnaire!
