# Supplementary material for: Profiling trial burden and patients’ attitudes to improve clinical research in epidermolysis bullosa
Source: Orphanet J Rare Dis. 2020 Jul 10;15:182. doi: 10.1186/s13023-020-01443-3 (PMC7350741; doi:10.1186/s13023-020-01443-3)
Supplement: Supplementary file 2 — Additional file 2: Supplementary Fig. 2a-c. Subgroup results – mild versus severe. Graphical representation of subgroup responses referring to diseases severity (patients with mild EB versus patients with severe EB). The numbers in the columns represent respondents for each option. By combining Likert scala points 1 and 2 as well as 4 and 5 we identified significant relations between disease severity and responses to three questions (*): a) The “desire for better treatment options” was higher in the severe group (73.3% vs 30.8%, p = 0.030); b) “Study visits can be organized via telemedicine or telephone” is more important for the mild group (90.0% vs 46.7%, p = 0.024); c) The “extent of scheduled invasive investigations (e.g. blood taking, biopsy)” that is a more important argument against participation for the severe subgroup (46.7% vs 7.7%, 0 = 0.029). Arguments for and against participation in a study were sorted by the subgroup’s total mean values in descending order. [file 13023_2020_1443_MOESM2_ESM.pptx]

## Slide 1
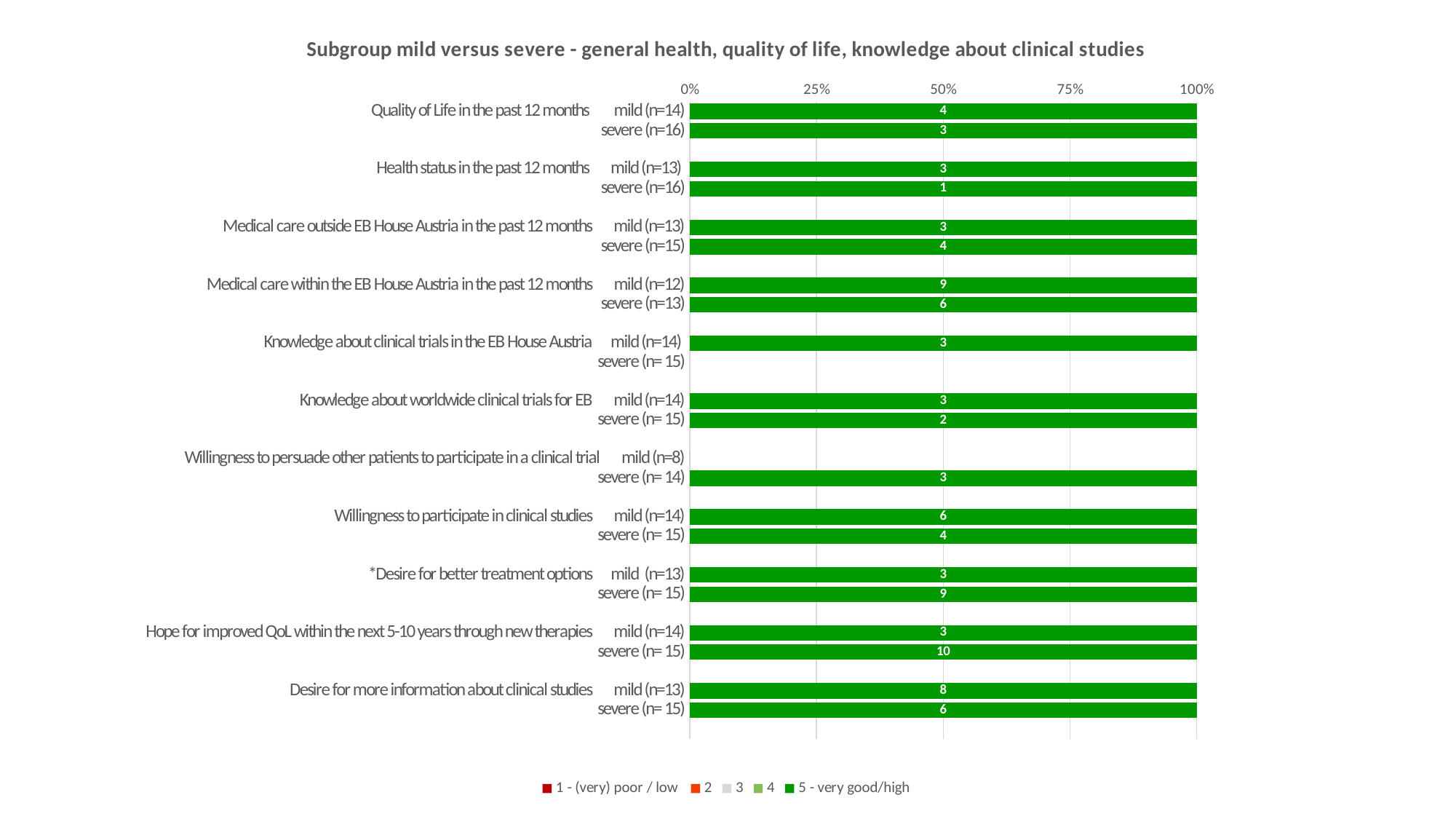

### Chart: Subgroup mild versus severe - general health, quality of life, knowledge about clinical studies
| Category | | | | | |
|---|---|---|---|---|---|
| Quality of Life in the past 12 months mild (n=14) | None | 1.0 | 1.0 | 8.0 | 4.0 |
| severe (n=16) | None | None | 4.0 | 9.0 | 3.0 |
| | None | None | None | None | None |
| Health status in the past 12 months mild (n=13) | None | None | 2.0 | 8.0 | 3.0 |
| severe (n=16) | None | 1.0 | 7.0 | 7.0 | 1.0 |
| | None | None | None | None | None |
| Medical care outside EB House Austria in the past 12 months mild (n=13) | 1.0 | None | 3.0 | 6.0 | 3.0 |
| severe (n=15) | None | 2.0 | 3.0 | 6.0 | 4.0 |
| | None | None | None | None | None |
| Medical care within the EB House Austria in the past 12 months mild (n=12) | None | None | None | 3.0 | 9.0 |
| severe (n=13) | None | None | 1.0 | 6.0 | 6.0 |
| | None | None | None | None | None |
| Knowledge about clinical trials in the EB House Austria mild (n=14) | 1.0 | 2.0 | 4.0 | 4.0 | 3.0 |
| severe (n= 15) | 2.0 | 1.0 | 6.0 | 6.0 | None |
| | None | None | None | None | None |
| Knowledge about worldwide clinical trials for EB mild (n=14) | 1.0 | 3.0 | 4.0 | 3.0 | 3.0 |
| severe (n= 15) | 1.0 | 2.0 | 4.0 | 6.0 | 2.0 |
| | None | None | None | None | None |
| Willingness to persuade other patients to participate in a clinical trial mild (n=8) | 1.0 | 0.0 | 4.0 | 3.0 | 0.0 |
| severe (n= 14) | 1.0 | 2.0 | 5.0 | 3.0 | 3.0 |
| | None | None | None | None | None |
| Willingness to participate in clinical studies mild (n=14) | 2.0 | 1.0 | 2.0 | 3.0 | 6.0 |
| severe (n= 15) | 3.0 | 2.0 | 2.0 | 4.0 | 4.0 |
| | None | None | None | None | None |
| *Desire for better treatment options mild (n=13) | 1.0 | 1.0 | 7.0 | 1.0 | 3.0 |
| severe (n= 15) | None | 2.0 | 2.0 | 2.0 | 9.0 |
| | None | None | None | None | None |
| Hope for improved QoL within the next 5-10 years through new therapies mild (n=14) | 2.0 | None | 3.0 | 6.0 | 3.0 |
| severe (n= 15) | 1.0 | 1.0 | 3.0 | None | 10.0 |
| | None | None | None | None | None |
| Desire for more information about clinical studies mild (n=13) | 1.0 | 1.0 | 2.0 | 1.0 | 8.0 |
| severe (n= 15) | None | 1.0 | 3.0 | 5.0 | 6.0 |

## Slide 2
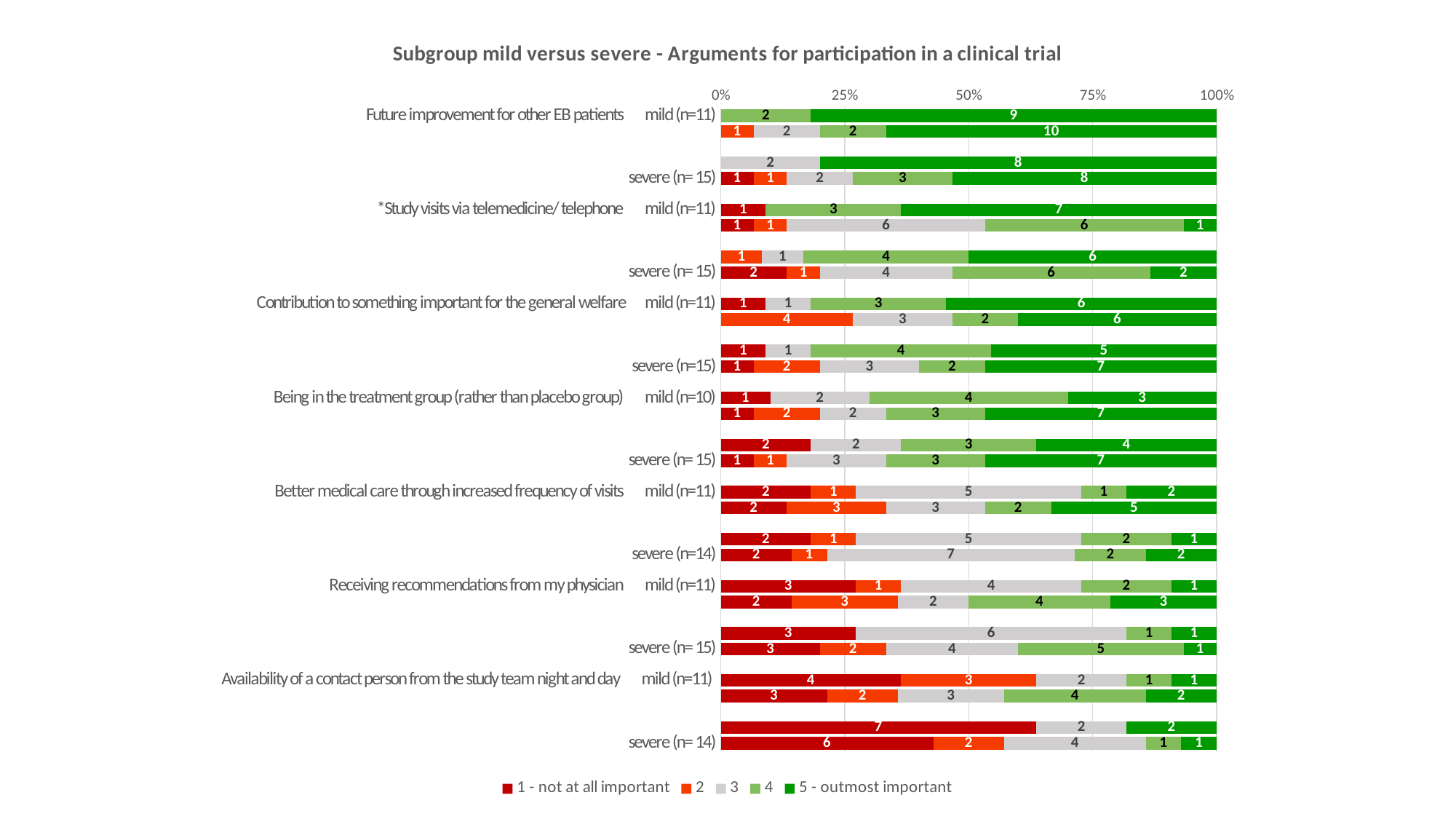

### Chart: Subgroup mild versus severe - Arguments for participation in a clinical trial
| Category | | | | | |
|---|---|---|---|---|---|
| Future improvement for other EB patients mild (n=11) | None | None | None | 2.0 | 9.0 |
| severe (n= 15) | None | 1.0 | 2.0 | 2.0 | 10.0 |
| | None | None | None | None | None |
| Alleviation of own symptoms mild (n=10) | None | None | 2.0 | None | 8.0 |
| severe (n= 15) | 1.0 | 1.0 | 2.0 | 3.0 | 8.0 |
| | None | None | None | None | None |
| *Study visits via telemedicine/ telephone mild (n=11) | 1.0 | None | None | 3.0 | 7.0 |
| severe (n= 15) | 1.0 | 1.0 | 6.0 | 6.0 | 1.0 |
| | None | None | None | None | None |
| Study visit times consider patients' need and are flexible mild (n=12) | None | 1.0 | 1.0 | 4.0 | 6.0 |
| severe (n= 15) | 2.0 | 1.0 | 4.0 | 6.0 | 2.0 |
| | None | None | None | None | None |
| Contribution to something important for the general welfare mild (n=11) | 1.0 | None | 1.0 | 3.0 | 6.0 |
| severe (n= 15) | None | 4.0 | 3.0 | 2.0 | 6.0 |
| | None | None | None | None | None |
| Contribute to an increased knowledge about the disease mild (n=11) | 1.0 | None | 1.0 | 4.0 | 5.0 |
| severe (n=15) | 1.0 | 2.0 | 3.0 | 2.0 | 7.0 |
| | None | None | None | None | None |
| Being in the treatment group (rather than placebo group) mild (n=10) | 1.0 | None | 2.0 | 4.0 | 3.0 |
| severe (n= 15) | 1.0 | 2.0 | 2.0 | 3.0 | 7.0 |
| | None | None | None | None | None |
| No additional expenses occur for participiants (e.g. for travelling) mild (n=11) | 2.0 | None | 2.0 | 3.0 | 4.0 |
| severe (n= 15) | 1.0 | 1.0 | 3.0 | 3.0 | 7.0 |
| | None | None | None | None | None |
| Better medical care through increased frequency of visits mild (n=11) | 2.0 | 1.0 | 5.0 | 1.0 | 2.0 |
| severe (n= 15) | 2.0 | 3.0 | 3.0 | 2.0 | 5.0 |
| | None | None | None | None | None |
| Receiving recommendations from social network or online fores mild (n=11) | 2.0 | 1.0 | 5.0 | 2.0 | 1.0 |
| severe (n=14) | 2.0 | 1.0 | 7.0 | 2.0 | 2.0 |
| | None | None | None | None | None |
| Receiving recommendations from my physician mild (n=11) | 3.0 | 1.0 | 4.0 | 2.0 | 1.0 |
| severe (n= 14) | 2.0 | 3.0 | 2.0 | 4.0 | 3.0 |
| | None | None | None | None | None |
| Receiving recommendations from friends mild (n=11) | 3.0 | None | 6.0 | 1.0 | 1.0 |
| severe (n= 15) | 3.0 | 2.0 | 4.0 | 5.0 | 1.0 |
| | None | None | None | None | None |
| Availability of a contact person from the study team night and day mild (n=11) | 4.0 | 3.0 | 2.0 | 1.0 | 1.0 |
| severe (n= 14) | 3.0 | 2.0 | 3.0 | 4.0 | 2.0 |
| | None | None | None | None | None |
| Attractive rewards (e.g. ipad, camera) are offered mild (n=11) | 7.0 | None | 2.0 | None | 2.0 |
| severe (n= 14) | 6.0 | 2.0 | 4.0 | 1.0 | 1.0 |

## Slide 3
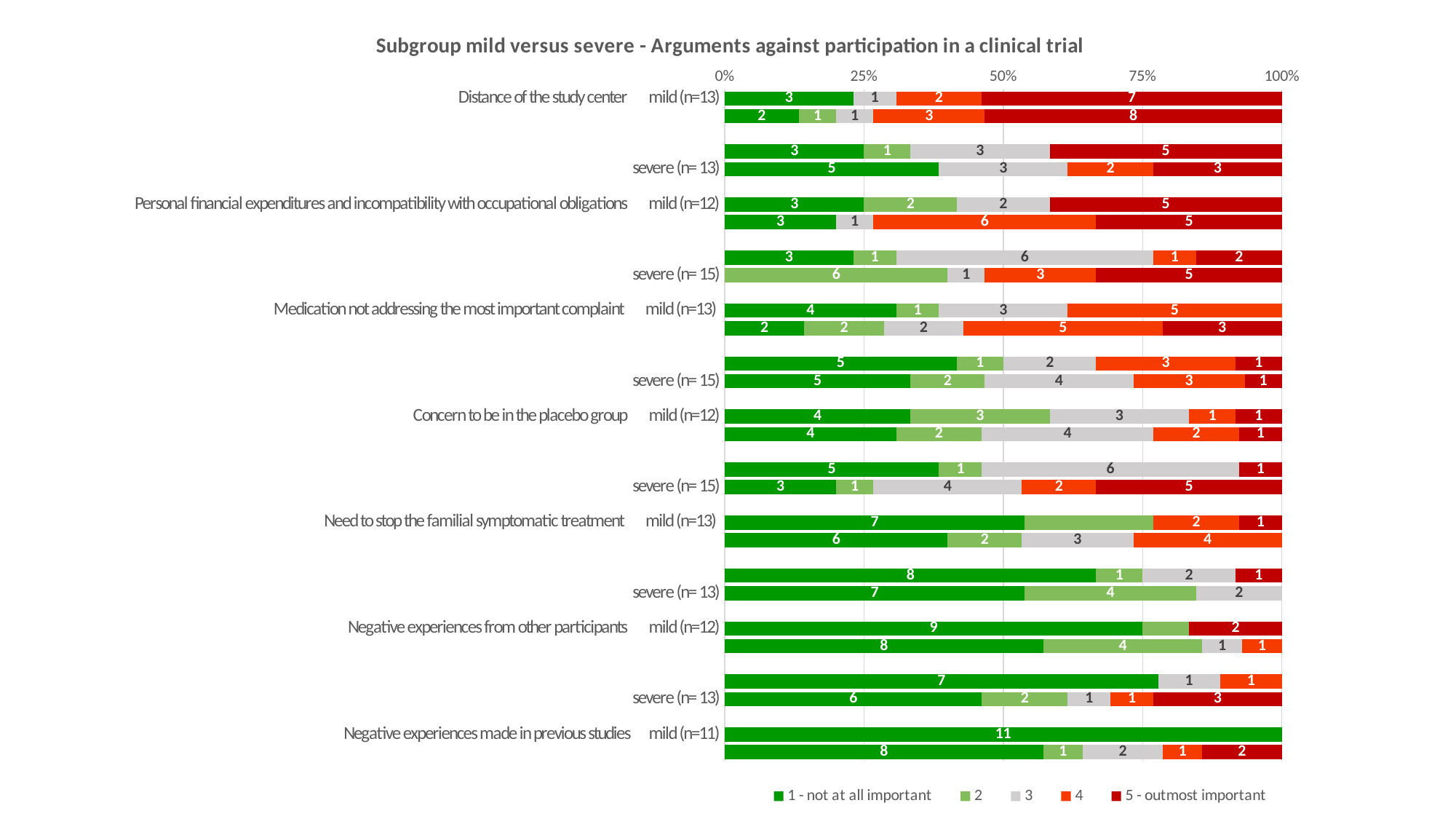

### Chart: Subgroup mild versus severe - Arguments against participation in a clinical trial
| Category | | | | | |
|---|---|---|---|---|---|
| Distance of the study center mild (n=13) | 3.0 | None | 1.0 | 2.0 | 7.0 |
| severe (n= 15) | 2.0 | 1.0 | 1.0 | 3.0 | 8.0 |
| | None | None | None | None | None |
| Failure to meet the inclusion criteria mild (n=12) | 3.0 | 1.0 | 3.0 | None | 5.0 |
| severe (n= 13) | 5.0 | None | 3.0 | 2.0 | 3.0 |
| | None | None | None | None | None |
| Personal financial expenditures and incompatibility with occupational obligations mild (n=12) | 3.0 | 2.0 | 2.0 | None | 5.0 |
| severe (n= 15) | 3.0 | None | 1.0 | 6.0 | 5.0 |
| | None | None | None | None | None |
| *Extent of possible adverse reactions or unknown risk of the study medication mild (n=13) | 3.0 | 1.0 | 6.0 | 1.0 | 2.0 |
| severe (n= 15) | None | 6.0 | 1.0 | 3.0 | 5.0 |
| | None | None | None | None | None |
| Medication not addressing the most important complaint mild (n=13) | 4.0 | 1.0 | 3.0 | 5.0 | None |
| severe (n= 14) | 2.0 | 2.0 | 2.0 | 5.0 | 3.0 |
| | None | None | None | None | None |
| Need to daily document complaints mild (n=12) | 5.0 | 1.0 | 2.0 | 3.0 | 1.0 |
| severe (n= 15) | 5.0 | 2.0 | 4.0 | 3.0 | 1.0 |
| | None | None | None | None | None |
| Concern to be in the placebo group mild (n=12) | 4.0 | 3.0 | 3.0 | 1.0 | 1.0 |
| severe (n= 13) | 4.0 | 2.0 | 4.0 | 2.0 | 1.0 |
| | None | None | None | None | None |
| *Extent of scheduled invasive investigations (e.g. blood taking, biopsy) mild (n=13) | 5.0 | 1.0 | 6.0 | None | 1.0 |
| severe (n= 15) | 3.0 | 1.0 | 4.0 | 2.0 | 5.0 |
| | None | None | None | None | None |
| Need to stop the familial symptomatic treatment mild (n=13) | 7.0 | 3.0 | None | 2.0 | 1.0 |
| severe (n= 14) | 6.0 | 2.0 | 3.0 | 4.0 | None |
| | None | None | None | None | None |
| Extent and comprehensibility of informed consent paper mild (n=12) | 8.0 | 1.0 | 2.0 | None | 1.0 |
| severe (n= 13) | 7.0 | 4.0 | 2.0 | None | 0.0 |
| | None | None | None | None | None |
| Negative experiences from other participants mild (n=12) | 9.0 | 1.0 | None | None | 2.0 |
| severe (n= 14) | 8.0 | 4.0 | 1.0 | 1.0 | None |
| | None | None | None | None | None |
| Adverse personal circumstances mild (n=9) | 7.0 | None | 1.0 | 1.0 | None |
| severe (n= 13) | 6.0 | 2.0 | 1.0 | 1.0 | 3.0 |
| | None | None | None | None | None |
| Negative experiences made in previous studies mild (n=11) | 11.0 | 0.0 | None | None | 0.0 |
| severe (n= 14) | 8.0 | 1.0 | 2.0 | 1.0 | 2.0 |
