# Supplementary material for: Profiling trial burden and patients’ attitudes to improve clinical research in epidermolysis bullosa
Source: Orphanet J Rare Dis. 2020 Jul 10;15:182. doi: 10.1186/s13023-020-01443-3 (PMC7350741; doi:10.1186/s13023-020-01443-3)
Supplement: Supplementary file 3 — Additional file 3: Supplementary Fig. 3a-c. Subgroup results - young (0–17 years of age) versus old (≥18 years of age). Graphical representation of the responses of age-subgroups (patients 0–17 years and patients 18 years of age or older). The numbers in the columns represent respondents for each option. By combining Likert scala points 1 and 2 as well as 4 and 5, we found that younger patients had a significant higher desire for better treatment options (75.0% vs 35.3%, p = 0.041), were significantly less averse to participate (8.3% [1/12] vs 44.4% [8/18], p = 0.040) and rated “the failure to meet inclusion criteria” a significantly less important barrier (10.0% vs 62.5%, p = 0.011)(*). [file 13023_2020_1443_MOESM3_ESM.pptx]

## Slide 1
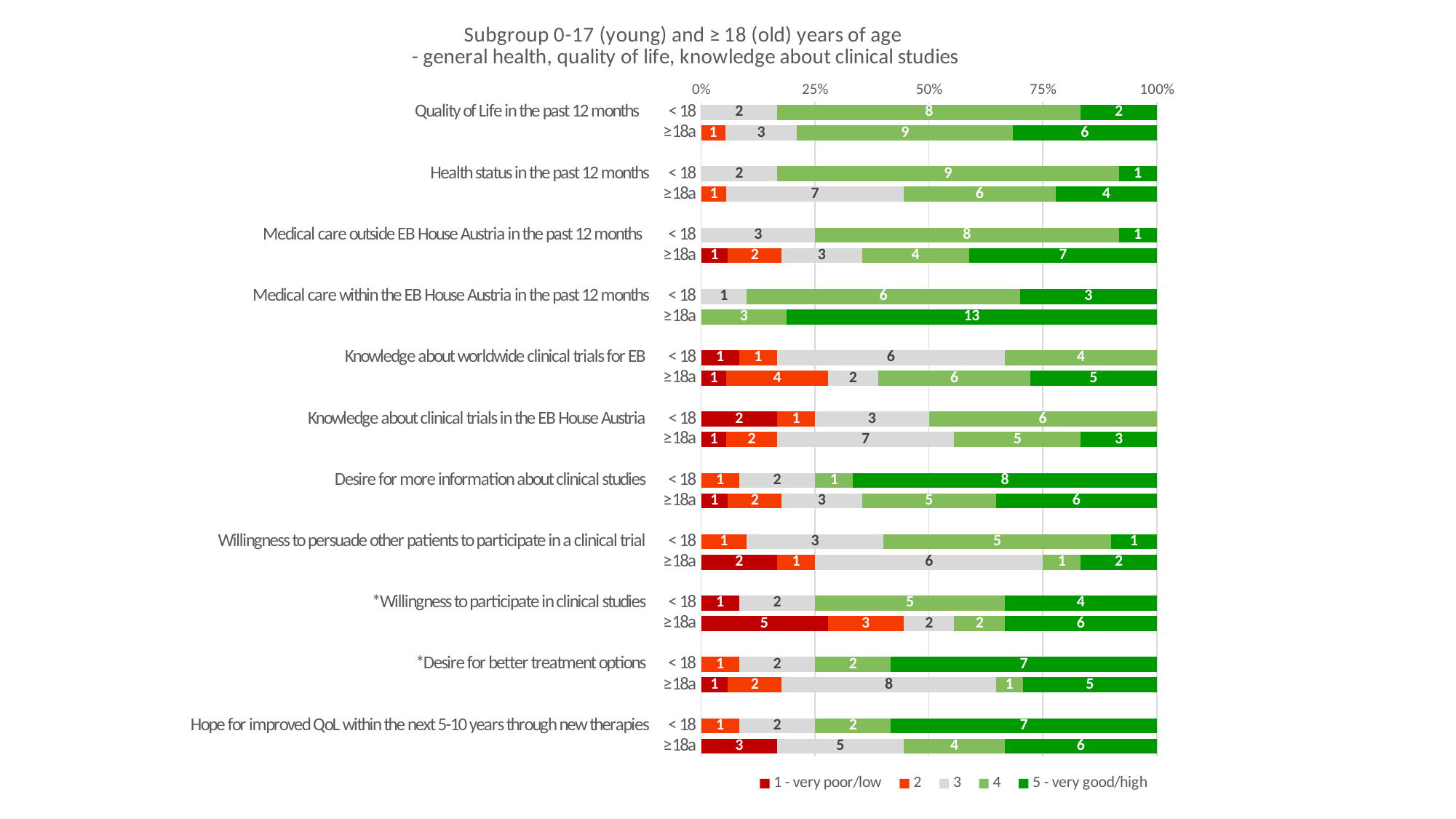

### Chart: Subgroup 0-17 (young) and ≥ 18 (old) years of age
 - general health, quality of life, knowledge about clinical studies
| Category | | | | | |
|---|---|---|---|---|---|
| Quality of Life in the past 12 months < 18 | 0.0 | None | 2.0 | 8.0 | 2.0 |
| ≥18a | None | 1.0 | 3.0 | 9.0 | 6.0 |
| | None | None | None | None | None |
| Health status in the past 12 months < 18 | None | None | 2.0 | 9.0 | 1.0 |
| ≥18a | None | 1.0 | 7.0 | 6.0 | 4.0 |
| | None | None | None | None | None |
| Medical care outside EB House Austria in the past 12 months < 18 | None | None | 3.0 | 8.0 | 1.0 |
| ≥18a | 1.0 | 2.0 | 3.0 | 4.0 | 7.0 |
| | None | None | None | None | None |
| Medical care within the EB House Austria in the past 12 months < 18 | None | None | 1.0 | 6.0 | 3.0 |
| ≥18a | None | None | None | 3.0 | 13.0 |
| | None | None | None | None | None |
| Knowledge about worldwide clinical trials for EB < 18 | 1.0 | 1.0 | 6.0 | 4.0 | None |
| ≥18a | 1.0 | 4.0 | 2.0 | 6.0 | 5.0 |
| | None | None | None | None | None |
| Knowledge about clinical trials in the EB House Austria < 18 | 2.0 | 1.0 | 3.0 | 6.0 | None |
| ≥18a | 1.0 | 2.0 | 7.0 | 5.0 | 3.0 |
| | None | None | None | None | None |
| Desire for more information about clinical studies < 18 | None | 1.0 | 2.0 | 1.0 | 8.0 |
| ≥18a | 1.0 | 2.0 | 3.0 | 5.0 | 6.0 |
| | None | None | None | None | None |
| Willingness to persuade other patients to participate in a clinical trial < 18 | None | 1.0 | 3.0 | 5.0 | 1.0 |
| ≥18a | 2.0 | 1.0 | 6.0 | 1.0 | 2.0 |
| | None | None | None | None | None |
| *Willingness to participate in clinical studies < 18 | 1.0 | None | 2.0 | 5.0 | 4.0 |
| ≥18a | 5.0 | 3.0 | 2.0 | 2.0 | 6.0 |
| | None | None | None | None | None |
| *Desire for better treatment options < 18 | None | 1.0 | 2.0 | 2.0 | 7.0 |
| ≥18a | 1.0 | 2.0 | 8.0 | 1.0 | 5.0 |
| | None | None | None | None | None |
| Hope for improved QoL within the next 5-10 years through new therapies < 18 | None | 1.0 | 2.0 | 2.0 | 7.0 |
| ≥18a | 3.0 | None | 5.0 | 4.0 | 6.0 |

## Slide 2
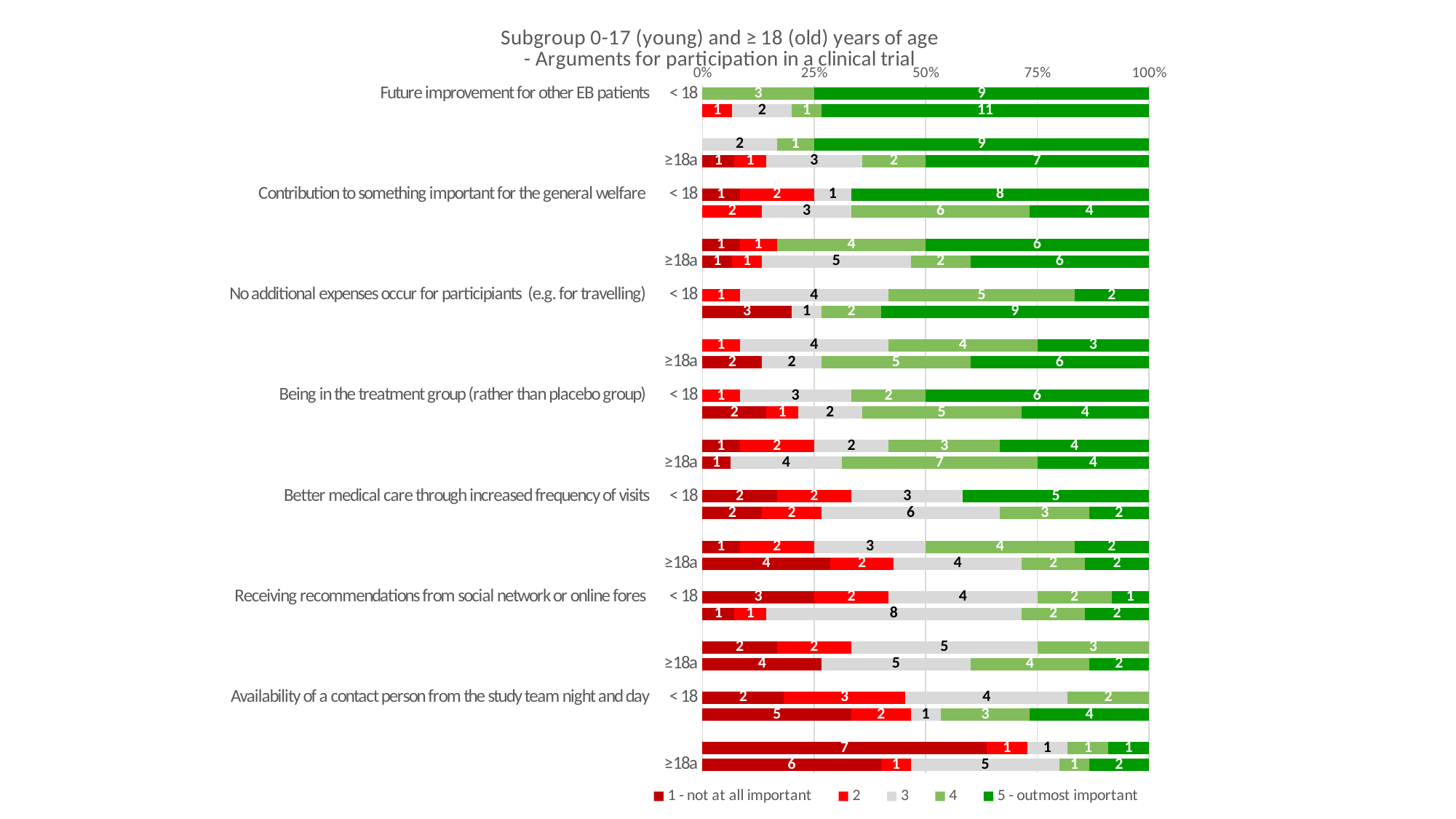

### Chart: Subgroup 0-17 (young) and ≥ 18 (old) years of age
- Arguments for participation in a clinical trial
| Category | | | | | |
|---|---|---|---|---|---|
| Future improvement for other EB patients < 18 | None | None | None | 3.0 | 9.0 |
| ≥18a | None | 1.0 | 2.0 | 1.0 | 11.0 |
| | None | None | None | None | None |
| Alleviation of own symptoms < 18 | None | None | 2.0 | 1.0 | 9.0 |
| ≥18a | 1.0 | 1.0 | 3.0 | 2.0 | 7.0 |
| | None | None | None | None | None |
| Contribution to something important for the general welfare < 18 | 1.0 | 2.0 | 1.0 | None | 8.0 |
| ≥18a | None | 2.0 | 3.0 | 6.0 | 4.0 |
| | None | None | None | None | None |
| Contribute to an increased knowledge about the disease < 18 | 1.0 | 1.0 | None | 4.0 | 6.0 |
| ≥18a | 1.0 | 1.0 | 5.0 | 2.0 | 6.0 |
| | None | None | None | None | None |
| No additional expenses occur for participiants (e.g. for travelling) < 18 | None | 1.0 | 4.0 | 5.0 | 2.0 |
| ≥18a | 3.0 | None | 1.0 | 2.0 | 9.0 |
| | None | None | None | None | None |
| Study visits via telemedicine/ telephone < 18 | None | 1.0 | 4.0 | 4.0 | 3.0 |
| ≥18a | 2.0 | None | 2.0 | 5.0 | 6.0 |
| | None | None | None | None | None |
| Being in the treatment group (rather than placebo group) < 18 | None | 1.0 | 3.0 | 2.0 | 6.0 |
| ≥18a | 2.0 | 1.0 | 2.0 | 5.0 | 4.0 |
| | None | None | None | None | None |
| Study visit times consider patients' need and are flexible < 18 | 1.0 | 2.0 | 2.0 | 3.0 | 4.0 |
| ≥18a | 1.0 | None | 4.0 | 7.0 | 4.0 |
| | None | None | None | None | None |
| Better medical care through increased frequency of visits < 18 | 2.0 | 2.0 | 3.0 | None | 5.0 |
| ≥18a | 2.0 | 2.0 | 6.0 | 3.0 | 2.0 |
| | None | None | None | None | None |
| Receiving recommendations from my physician < 18 | 1.0 | 2.0 | 3.0 | 4.0 | 2.0 |
| ≥18a | 4.0 | 2.0 | 4.0 | 2.0 | 2.0 |
| | None | None | None | None | None |
| Receiving recommendations from social network or online fores < 18 | 3.0 | 2.0 | 4.0 | 2.0 | 1.0 |
| ≥18a | 1.0 | 1.0 | 8.0 | 2.0 | 2.0 |
| | None | None | None | None | None |
| Receiving recommendations from friends < 18 | 2.0 | 2.0 | 5.0 | 3.0 | None |
| ≥18a | 4.0 | None | 5.0 | 4.0 | 2.0 |
| | None | None | None | None | None |
| Availability of a contact person from the study team night and day < 18 | 2.0 | 3.0 | 4.0 | 2.0 | None |
| ≥18a | 5.0 | 2.0 | 1.0 | 3.0 | 4.0 |
| | None | None | None | None | None |
| Attractive rewards (e.g. ipad, camera) are offered < 18 | 7.0 | 1.0 | 1.0 | 1.0 | 1.0 |
| ≥18a | 6.0 | 1.0 | 5.0 | 1.0 | 2.0 |

## Slide 3
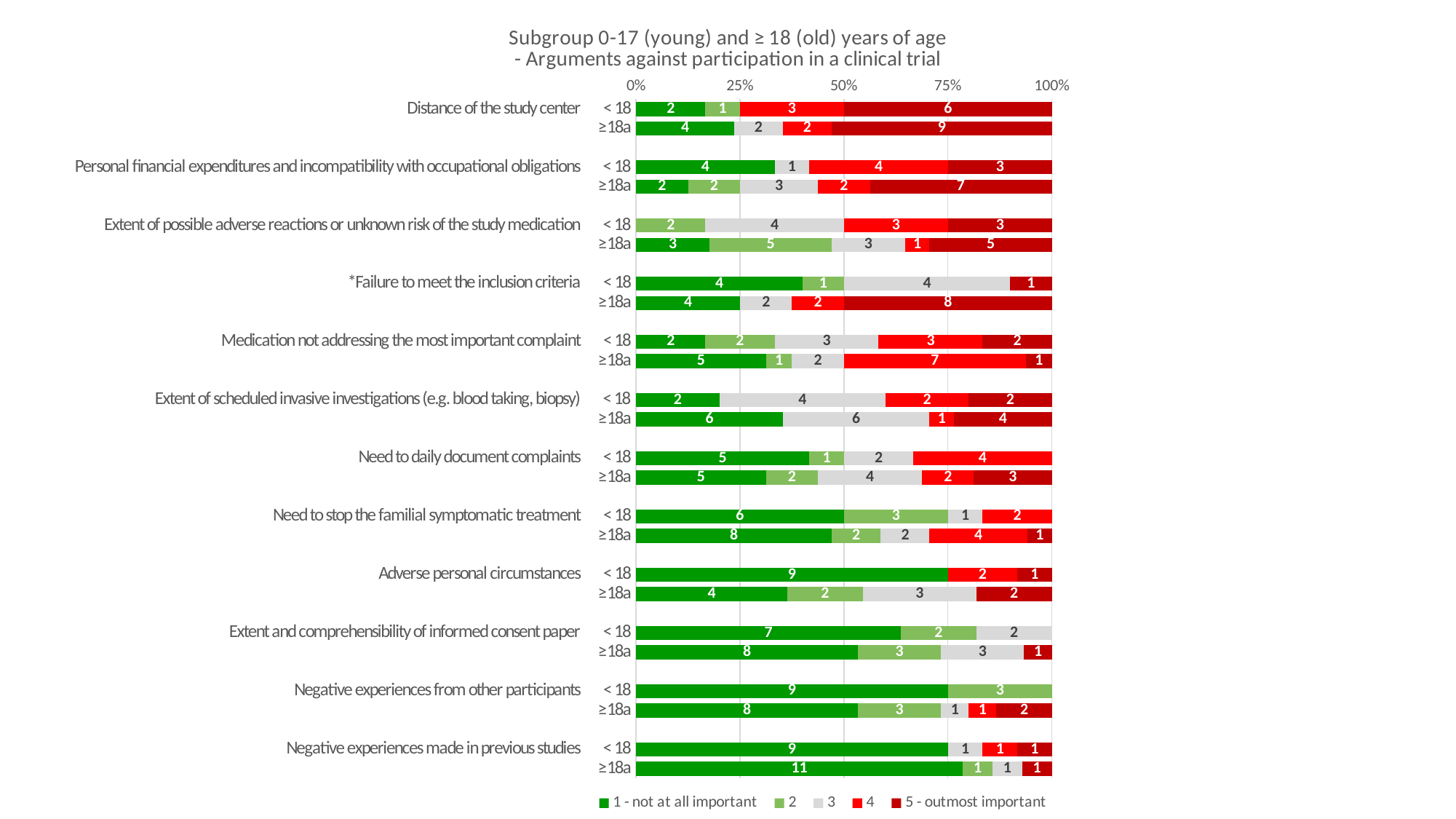

### Chart: Subgroup 0-17 (young) and ≥ 18 (old) years of age
- Arguments against participation in a clinical trial
| Category | | | | | |
|---|---|---|---|---|---|
| Distance of the study center < 18 | 2.0 | 1.0 | None | 3.0 | 6.0 |
| ≥18a | 4.0 | None | 2.0 | 2.0 | 9.0 |
| | None | None | None | None | None |
| Personal financial expenditures and incompatibility with occupational obligations < 18 | 4.0 | None | 1.0 | 4.0 | 3.0 |
| ≥18a | 2.0 | 2.0 | 3.0 | 2.0 | 7.0 |
| | None | None | None | None | None |
| Extent of possible adverse reactions or unknown risk of the study medication < 18 | None | 2.0 | 4.0 | 3.0 | 3.0 |
| ≥18a | 3.0 | 5.0 | 3.0 | 1.0 | 5.0 |
| | None | None | None | None | None |
| *Failure to meet the inclusion criteria < 18 | 4.0 | 1.0 | 4.0 | None | 1.0 |
| ≥18a | 4.0 | None | 2.0 | 2.0 | 8.0 |
| | None | None | None | None | None |
| Medication not addressing the most important complaint < 18 | 2.0 | 2.0 | 3.0 | 3.0 | 2.0 |
| ≥18a | 5.0 | 1.0 | 2.0 | 7.0 | 1.0 |
| | None | None | None | None | None |
| Extent of scheduled invasive investigations (e.g. blood taking, biopsy) < 18 | 2.0 | None | 4.0 | 2.0 | 2.0 |
| ≥18a | 6.0 | None | 6.0 | 1.0 | 4.0 |
| | None | None | None | None | None |
| Need to daily document complaints < 18 | 5.0 | 1.0 | 2.0 | 4.0 | None |
| ≥18a | 5.0 | 2.0 | 4.0 | 2.0 | 3.0 |
| | None | None | None | None | None |
| Need to stop the familial symptomatic treatment < 18 | 6.0 | 3.0 | 1.0 | 2.0 | None |
| ≥18a | 8.0 | 2.0 | 2.0 | 4.0 | 1.0 |
| | None | None | None | None | None |
| Adverse personal circumstances < 18 | 9.0 | None | None | 2.0 | 1.0 |
| ≥18a | 4.0 | 2.0 | 3.0 | None | 2.0 |
| | None | None | None | None | None |
| Extent and comprehensibility of informed consent paper < 18 | 7.0 | 2.0 | 2.0 | None | None |
| ≥18a | 8.0 | 3.0 | 3.0 | None | 1.0 |
| | None | None | None | None | None |
| Negative experiences from other participants < 18 | 9.0 | 3.0 | None | None | None |
| ≥18a | 8.0 | 3.0 | 1.0 | 1.0 | 2.0 |
| | None | None | None | None | None |
| Negative experiences made in previous studies < 18 | 9.0 | None | 1.0 | 1.0 | 1.0 |
| ≥18a | 11.0 | 1.0 | 1.0 | None | 1.0 |
