# Supplementary material for: Profiling trial burden and patients’ attitudes to improve clinical research in epidermolysis bullosa
Source: Orphanet J Rare Dis. 2020 Jul 10;15:182. doi: 10.1186/s13023-020-01443-3 (PMC7350741; doi:10.1186/s13023-020-01443-3)
Supplement: Supplementary file 4 — Additional file 4: Supplementary Fig. 4 Maximum extent of individual expenses considered acceptable for participation in a clinical trial. Graphical presentation of part four of the survey, asking for the extent of individual expenses considered acceptable for participation in a clinical study. Mean values of the four subgroups (mild (patients with mild EB type), severe (patients with severe EB type), young (< 18 years of age), old (18 years of age or older)) are indicated in different colors. [file 13023_2020_1443_MOESM4_ESM.zip › Supplementary 4.docx]

|  | | **Total** | **Mild subgroup** | **Severe subgroup** | **Young subgroup (< 18 years)** | **Old subgroup (≥ 18 years)** |
| --- | --- | --- | --- | --- | --- | --- |
| Max. accepted travel time to the study center (in h) | Mean | **4.5** | **3.0** | **5.7** | **5.2** | **4.4** |
|  | N | 30.0 | 10.0 | 14.0 | 11.0 | 14.0 |
|  | SD | 3.7 | 3.0 | 4.6 | 3.3 | 4.6 |
| Max. tolerated frequency of study visits (every … weeks) | Mean | **5.5** | **4.6** | **6.2** | **7.0** | **4.2** |
|  | N | 29.0 | 9.0 | 14.0 | 11.0 | 13.0 |
|  | SD | 2.9 | 1.7 | 3.6 | 2.9 | 2.6 |
| Max. tolerated frequency of study-related dressing changes or application of creams (per day) | Mean | **1.2** | **1.2** | **1.2** | **1.2** | **1.1** |
|  | N | 28.0 | 9.0 | 13.0 | 10.0 | 13.0 |
|  | SD | 0.7 | 0.9 | 0.5 | 0.5 | 0.9 |
| Max. tolerated number of phone calls from the study team (per week) | Mean | **1.8** | **1.8** | **2.1** | **1.7** | **2.1** |
|  | N | 28.0 | 9.0 | 13.0 | 9.0 | 14.0 |
|  | SD | 1.1 | 1.1 | 1.3 | 1.0 | 1.2 |
| Max. tolerated frequency of blood taking (every … weeks) | Mean | **5.0** | **4.4** | **5.0** | **6.8** | **3.2** |
|  | N | 28.0 | 10.0 | 12.0 | 10.0 | 13.0 |
|  | SD | 2.8 | 1.9 | 3.2 | 2.4 | 1.3 |
| Max. tolerated frequency of skin biopsy (every … weeks) | Mean | **17.0** | **10.7** | **22.6** | **26.2** | **9.7** |
|  | N | 26.0 | 9.0 | 12.0 | 10.0 | 12.0 |
|  | SD | 23.5 | 8.8 | 31.2 | 30.7 | 13.8 |
| Max. duration of inpatient (overnight) stay (in days) | Mean | **3.4** | **1.8** | **5.6** | **3.0** | **4.4** |
|  | N | 25.0 | 9.0 | 11.0 | 9.0 | 12.0 |
|  | SD | 5.9 | 0.8 | 8.6 | 2.8 | 8.2 |
| Max. tolerated frequency of inpatient stay (every … weeks) | Mean | **14.5** | **12.5** | **15.1** | **19.9** | **9.8** |
|  | N | 24.0 | 9.0 | 10.0 | 9.0 | 11.0 |
|  | SD | 15.5 | 14.3 | 14.7 | 17.5 | 8.6 |

Abbr.: freq = frequency; h = hours;
