# Supplementary figures and images for: Profiling trial burden and patients’ attitudes to improve clinical research in epidermolysis bullosa
Source: Orphanet J Rare Dis. 2020 Jul 10;15:182. doi: 10.1186/s13023-020-01443-3 (PMC7350741; doi:10.1186/s13023-020-01443-3)

## Slide 1
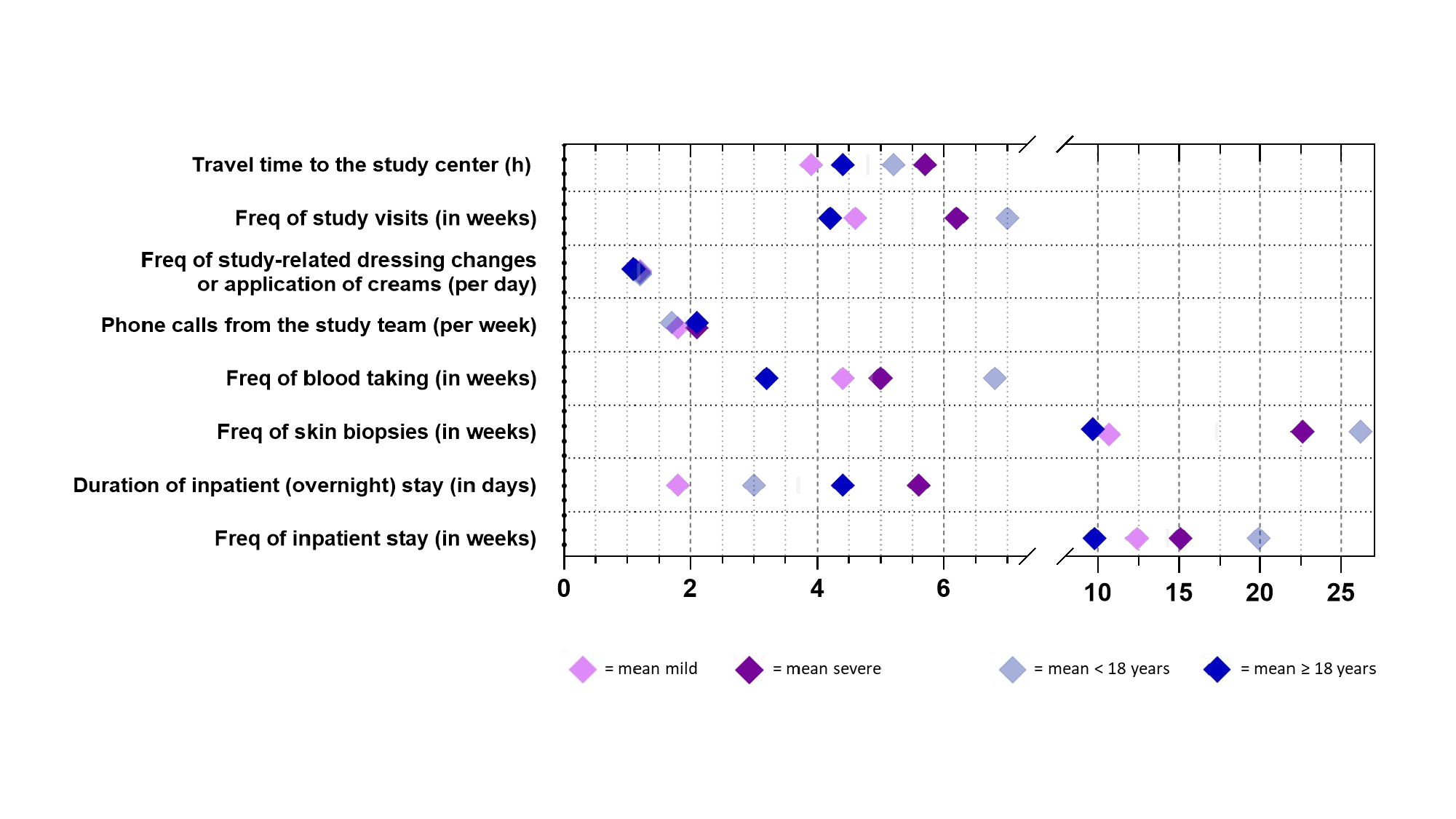

Supplement: Supplementary file 4 — Additional file 4: Supplementary Fig. 4 Maximum extent of individual expenses considered acceptable for participation in a clinical trial. Graphical presentation of part four of the survey, asking for the extent of individual expenses considered acceptable for participation in a clinical study. Mean values of the four subgroups (mild (patients with mild EB type), severe (patients with severe EB type), young (< 18 years of age), old (18 years of age or older)) are indicated in different colors. [file 13023_2020_1443_MOESM4_ESM.zip › Supplementary Fig 4.pptx]
