# Supplementary material for: Profiling trial burden and patients’ attitudes to improve clinical research in epidermolysis bullosa
Source: Orphanet J Rare Dis. 2020 Jul 10;15:182. doi: 10.1186/s13023-020-01443-3 (PMC7350741; doi:10.1186/s13023-020-01443-3)
Supplement: Supplementary file 5 — Additional file 5: Supplementary Fig. 5. Main sources of knowledge about clinical studies. The main sources of knowledge about clinical studies in this study cohort were the EB-newsletter (https://www.debra-austria.org/newsletter) 63.9%, n = 23), the patient groups DEBRA Austria and Italy (61.1%, n = 22), the EB House Salzburg (47.2%, n = 17), internet (41.7%, n = 15), and annual DEBRA Austria meetings (13.9%, n = 3). Three participants (8.3%) stated to have lacked any sources. [file 13023_2020_1443_MOESM5_ESM.pptx]

## Slide 1
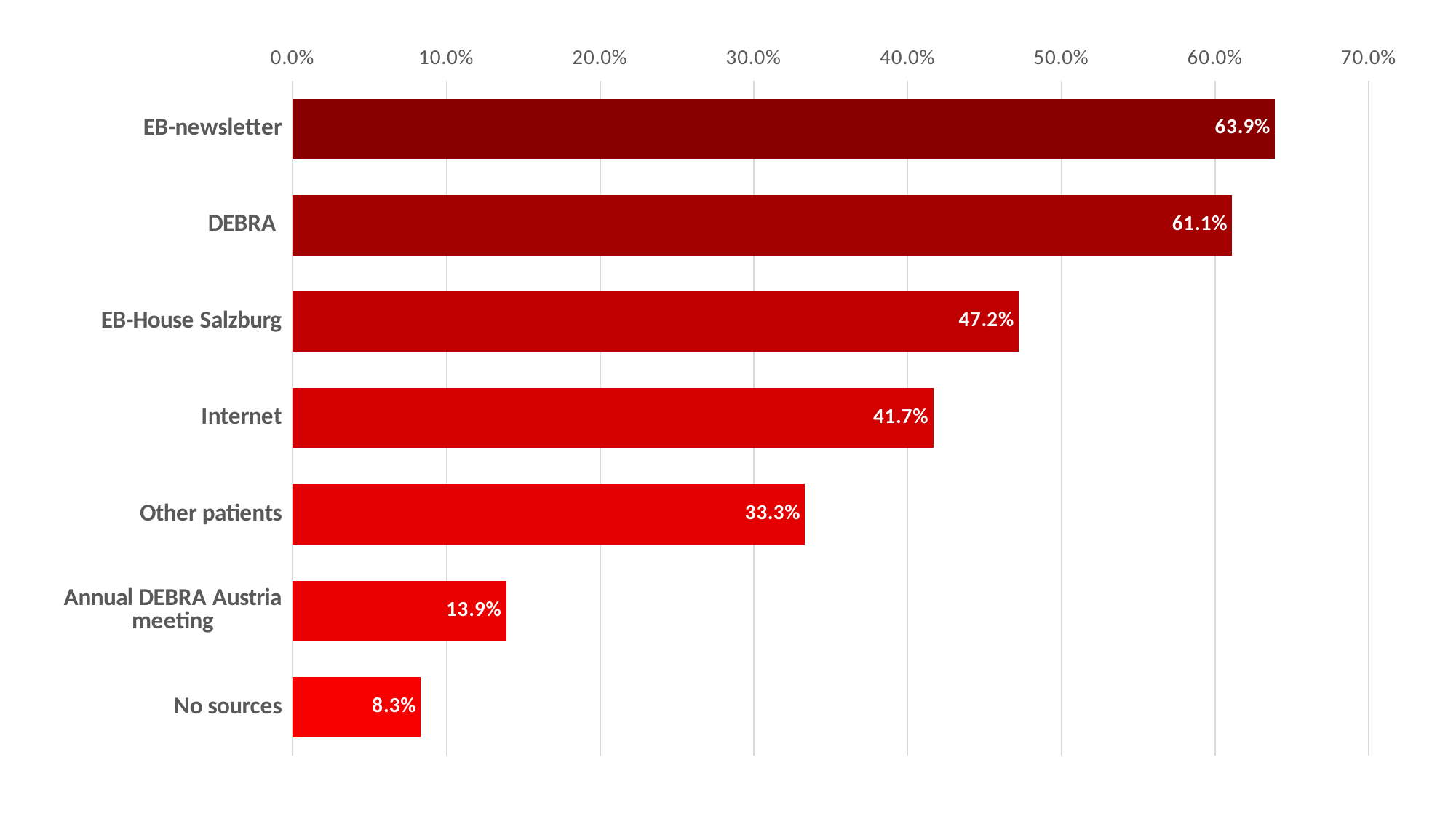

### Chart
| Category | |
|---|---|
| EB-newsletter | 0.6388888888888888 |
| DEBRA | 0.6111111111111112 |
| EB-House Salzburg | 0.4722222222222222 |
| Internet | 0.4166666666666667 |
| Other patients | 0.3333333333333333 |
| Annual DEBRA Austria meeting | 0.1388888888888889 |
| No sources | 0.08333333333333333 |
